# Teacups, a Python Package for the Simulation of Time-Resolved EPR Spectra of Spin-Polarized Multi-Spin Systems

**DOI:** 10.1021/acs.jpca.5c01512

**Published:** 2025-03-28

**Authors:** Theresia Quintes, Stefan Weber, Sabine Richert

**Affiliations:** Institute of Physical Chemistry, University of Freiburg, Albertstraße 21, 79104 Freiburg, Germany

## Abstract

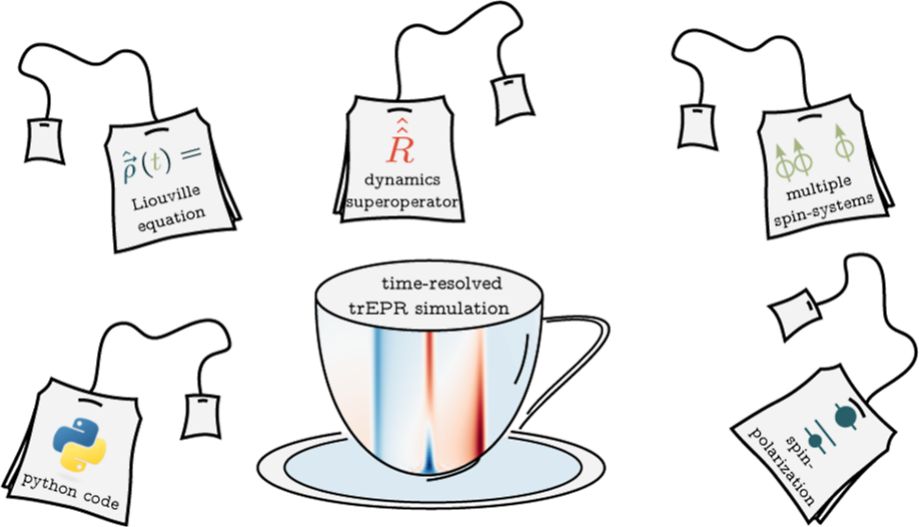

Spin-polarized magnetic systems, generated by the interaction of photoactive molecules with light, play a key role in a wide range of scientific applications. Representative examples are OLEDs, organic photovoltaics, and singlet fission. Further, they are important intermediates in certain biological processes including photosynthesis and, possibly, avian magnetoreception. Transient continuous-wave electron paramagnetic resonance (trEPR) spectroscopy is a powerful tool to reveal the temporal evolution of nonequilibrium spin states, which contains valuable information on any photoinduced dynamic processes occurring in these systems. For the analysis of the recorded trEPR data, simulations are essential. While the simulation of static trEPR spectra is supported well by tools like EasySpin, the simulation of time-resolved trEPR data is less developed. Here, we introduce teacups, a new freely available and well-documented Python-based routine for the simulation of the temporal evolution of trEPR spectra. The internal dynamics of different spin-polarized systems can be analyzed, thereby enhancing our mechanistic understanding. In this manuscript, we explain the theoretical background and provide a description of the features and setup of teacups. Further, a step-by-step example for data analysis is provided.

## 1 Introduction

Over the last decades, the study of spin-polarized magnetic systems has emerged as a captivating frontier.^1^ These systems, many of which are generated by the interaction of photoactive molecules with light, are special in the sense that their energy levels are selectively populated, leading to population differences far from thermal equilibrium. They play an important role in diverse fields of science and technology. For instance, light-induced triplet states are key intermediates in the processes occurring in certain molecular spintronic devices,^2^ organic light-emitting diodes (OLEDs) based on thermally activated delayed fluorescence (TADF),^3^ in photosynthesis,^4,5^ organic photovoltaics,^6,7^ and can be employed as versatile spin labels.^8−11^ Another important research area is the analysis of spin-correlated radical pairs. They are involved, e.g., in biological processes such as photosynthesis and, presumably, in avian magnetoreception and the circadian clock.^5,12−22^ Triplet pair states, generated by singlet fission, are intermediates in the photophysical processes occurring in OLEDs and photovoltaic devices.^23−26^ Further, the exploration of the use of paired triplet and doublet states as quantum bits (qubits) in quantum information science is a rather new field of research involving spin-polarized systems.^27−32^

For the analysis of spin-polarized systems, transient electron paramagnetic resonance (trEPR) spectroscopy is a powerful analytical tool.^33^ In the experiment, typically short-lived paramagnetic species are generated with a short laser pulse and the transient EPR response is detected in the presence of a weak microwave magnetic field. The acquired data reveal the temporal evolution of the formed spin states and ultimately yield deep insights into the internal dynamics. This includes coherent spin phenomena like transient nutations^34^ and quantum beats,^35^ as well as incoherent dynamics such as structural motion,^36^ all of which have been investigated previously.^33,37^

For the interpretation of complex EPR spectra, simulations are essential. Simulations of trEPR spectra under static conditions are often performed using the powerful simulation tool EasySpin,^37−39^ but the analysis of time-resolved trEPR data is not as well established. EasySpin has recently been extended to include further capabilities for the simulation of transient EPR data,^37^ but does currently not support the simulation of (i) coherence effects such as quantum beats, (ii) transient nutations (Torrey oscillations) arising from the precession of the magnetization about the microwave field, or (iii) advanced relaxation processes and diffusive dynamics.

Other tools for simulating time-resolved phenomena are available, but often focus on different detection schemes. As an example, the time-evolution of spin systems after application of a microwave pulse can be simulated using the Spidyan^39^ package (now included in EasySpin) or the Spinach package.^40^ For the simulation of transient pulse EPR data, Spinach includes all the required functionalities, but as it has been developed for NMR simulations and large spin systems, the setup is significantly more complex: Its use requires advanced knowledge in simulation theory, as users have to compose their own simulation routines from toolbox functions. Both EasySpin and Spinach have the disadvantage that they are dependent on the commercial software MATLAB.

In this work, we introduce the new Python-based simulation routine teacups (Time-resolved EPR: Algebraic calculation of unequally populated spin systems) for the analysis of trEPR spectra which is meant to overcome the current limitations of EasySpin regarding the simulation of the time-evolution of transient EPR data.

Time-resolved simulations of trEPR spectra as such, have already been employed in various previous studies,^34,35,41−55^ but the source codes of the utilized simulation routines are often either not freely available, not sufficiently documented, or only suitable for a specific spin system. In contrast, teacups aims to provide a straightforward, user-friendly implementation, that is useable for several spin systems, filling a gap in the current landscape. By providing well-documented open-source code and adopting the widely used EasySpin syntax, teacups ensures ease of use and accessibility.

In this manuscript, we present the theoretical background for the simulation of time-domain EPR data followed by a detailed description of the features and setup of the program. We include information for developers as well as potential users by providing a step-by-step example for an analysis of a trEPR spectrum using the teacups routine and illustrating different applications based on literature examples.

## 2 Theoretical Methods

### 2.1 Introduction to the Transient EPR Experiment

To understand the physical principles underlying the simulation of time-domain EPR data, some knowledge about the trEPR experiment is necessary as the teacups simulation routine generates a transient continuous wave EPR spectrum by modeling the experiment.

**Figure 1:**
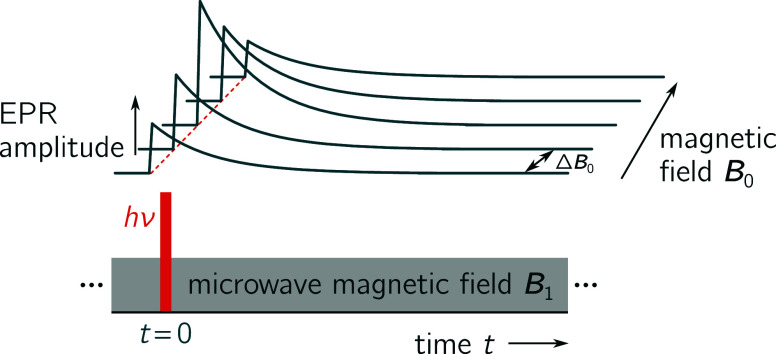
Schematic drawing of a transient EPR spectrum. The measurement is started shortly before the application of a nanosecond laser pulse. During the experiment the sample is continuously irradiated with microwaves (*B*_1_). At specific magnetic field points *B*_0_, kinetic traces are recorded. By accumulating data for multiple magnetic field points, a 3D data set is obtained. Adapted with permission from ref (33). Copyright 2025 John Wiley and Sons.

Transient EPR is a time-resolved EPR technique which is based on the standard continuous wave (cw) EPR experiment, for a recent review see.^33^ The EPR signal is detected directly without the application of any modulation, so that any positive signals correspond to microwave absorption and any negative signals to microwave emission. When running the experiment, the sample is placed into a static magnetic field and exposed to continuous microwave radiation. A nanosecond laser pulse is then applied and a kinetic trace recorded. Subsequently, the static magnetic field is changed and another kinetic trace is recorded. When repeating this for the entire magnetic field range of interest, a 3D spectrum is obtained, showing the time-behavior as a function of the static magnetic field (see Figure 1). The data thus contain information about the spin system as well as the excited state dynamics.^33^

### 2.2 Mathematical Modeling

**Figure 2:**
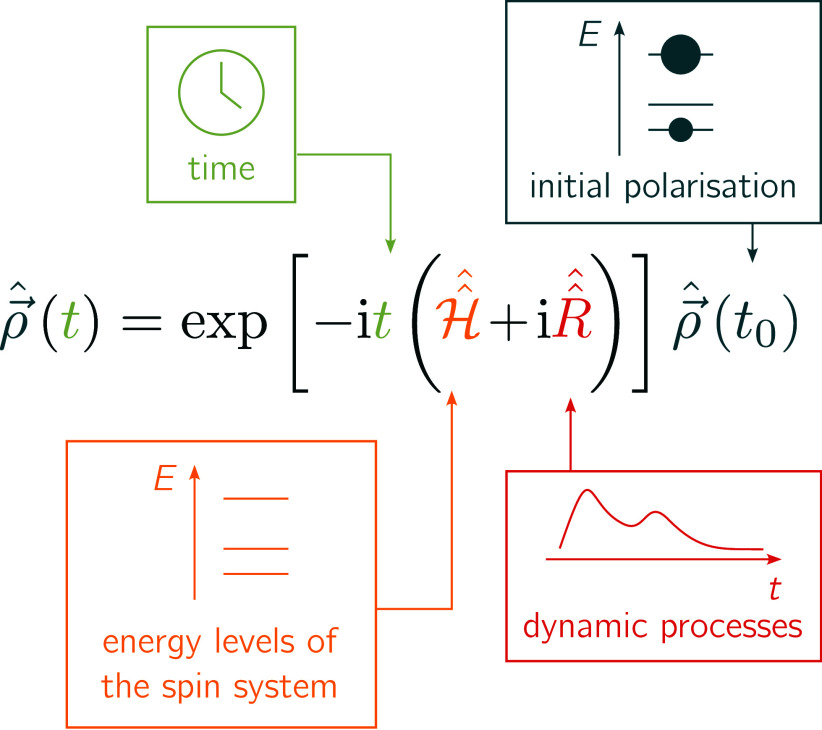
Solution of the stochastic Liouville equation. Multiple input parameters are needed for the calculation of the time-dependent density matrix ***ρ*^**. These are (i) the time point *t* for which the density matrix shall be calculated, (ii) the Hamiltonian superoperator  containing information on the spin system, (iii) the dynamics superoperator  containing information on the time-dependent transitions between the states, and (iv) the initial density matrix containing populations and coherences at time point *t*_0_. The resulting density matrix describes the longitudinal and transverse magnetization of the spin system at the time point *t*.

When trying to model the experimental conditions, we have to consider the spin system, the initial polarization after the laser pulse, and any time-dependent mechanisms involving transitions between the spin states. These three components need to be described mathematically and are fed into the solution of the stochastic Liouville equation^56−59^ as shown schematically in Figure 2. This equation allows us to calculate the polarization of the spin system as a function of the time *t* using multiple operators: The spin Hamiltonian , the density operator ***ρ*^**, and the time-evolution operator ***R*^**.^58,60−62^ In the following sections, the theoretical construction of a trEPR spectrum is described by explaining the significance of these operators.

### 2.3 Spin System

The spin system is defined by the number of spins and their magnetic interactions. Its energy levels are described by the Hamiltonian, which is set up as the sum of the Hamiltonians for all magnetic interactions
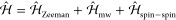
1

The interactions with the static magnetic field  and the time-dependent microwave field  are defined by the experimental conditions and are present for all spin systems investigated by trEPR. The spin interaction Hamiltonian  depends on the chosen spin system and includes all magnetic interactions between the spins. All operators are set up as matrices, which are summed up. They are obtained by matrix multiplication of the spin matrices (i.e., normalized Pauli matrices in units of *ℏ*), the magnetic fields, and the tensors which define the strength and direction of the interactions as explained in the following.

The interaction of the spin system with a static magnetic field is given by the sum of the electronic Zeeman interactions of all spins *i* with the magnetic field, scaled by the ***g***-tensor
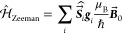
2where  is the spin vector operator  including the normalized Pauli matrices for each spin, and  the vector representing the magnetic field with a single nonzero component along the *z*-axis. Therefore, the equation can be simplified to
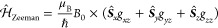
3where the scalar value for the magnetic field strength *B*_0_ and the elements of the ***g***-tensor *g*_*ij*_ are used.^61,63,64^

Since the microwave field (, with the amplitude *B*_1_) oscillates with time, the interaction with the microwave field (and following from that ) is time-dependent. However, the stochastic Liouville equation can only be solved for time-independent Hamiltonians. To address this, one must either ensure that the Hamiltonian remains time-independent or resort to piecewise integration over time-constant intervals, which introduces considerable computational complexity.^62^

To obtain a time-independent Hamiltonian, the coordinate system is transferred to the rotating frame by adding a frequency offset to the Zeeman frequencies^63^
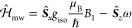
4

The rotating frame rotates with the microwave frequency ω so that the microwave Hamiltonian becomes time-independent. As the microwave field is applied perpendicular to the static magnetic field, the Hamiltonian represents the interaction of the *x*-part of all spins with the microwave field.

It needs to be considered that all Hamiltonians, that are nondiagonal, will become time-dependent in the rotating frame. To avoid this, the secular approximation is applied (as shown in more detail further below in Section 3), which is common practice in EPR spectroscopy. This approximation makes the interaction Hamiltonians diagonal, ensuring that they remain time-independent in the rotating frame. To demonstrate that this approach is valid even for highly anisotropic systems, we compare static EPR spectra computed using our simulation routine with those calculated using EasySpin in the Supporting Information (see Figure S1).^63^

In summary, by employing the secular approximation in combination with the rotating frame, the Hamiltonian becomes time-independent, which makes it possible to use the solution of the stochastic Liouville differential equation for our calculations.

In the following, a simple two-state-system (e.g., a single electron placed into a magnetic field) is assumed. The Hamiltonian for the chosen two-state example system consists only of the interactions of one electron (*s* = 1/2) with the magnetic field. The interaction matrix is given by
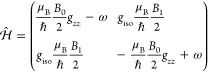
5

If the system consists of multiple spins, further interactions must be taken into account. These are the exchange interaction scaled by the exchange interaction parameter *J*, and the dipolar interactions between the spins scaled by a dipolar interaction tensor ***D***
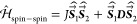
6Here,  and  are the spin vector operators of two coupled spin systems.^63−65^ Common examples are two electrons spins forming a radical pair, the two electron spins of a photogenerated triplet state (where the dipolar interaction becomes the zero-field splitting interaction and *J* is extremely large), or a triplet state coupled to a third electron spin.^37,48,66^ Further details are given in Section 3.

### 2.4 Polarization

For a light-induced system, as monitored in a trEPR experiment, the populations of the spin states deviate from thermal (Boltzmann) population and depend on the mechanism underlying the formation of the excited species. The resulting spectrum is said to be spin-polarized and typically characterized by the observation of absorptive as well as emissive signals, in contrast to the purely absorptive features observed in the Boltzmann case.^33,37^ The systems magnetization is described mathematically by the density matrix operator. The density operator ***ρ*^** contains the projections of the states |Ψ⟩ and describes a density distribution. For the two-state system introduced above (with two energetic eigenstates split by the Zeeman interaction, see eq 5), it takes the following general form
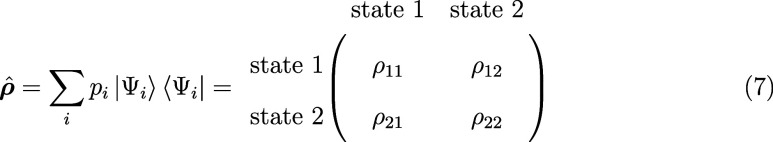
7where the diagonal elements represent the probability to find the system in the considered state, i.e. the populations of the states. The population differences result in the longitudinal magnetization (along the *z*-axis).^67^ If an energetically lower state is overpopulated, this leads to an absorptive spectral transition, whereas an overpopulation of a higher-lying energetic state results in an emissive transition. The off-diagonal elements are the coherences and connect the states. If they are different from zero, this corresponds to a transverse magnetization (in the *xz*-plane). At time *t* = 0, the off-diagonal elements are set to zero for the simulation. It is assumed that population differences are only created by the application of the laser pulse. By applying the microwave Hamiltonian, the off-diagonals become populated over time. This creates the detectable transverse magnetization.^62,63,68^

### 2.5 Time-Evolution

With the Hamiltonian and the density matrix at hand, the system can be fully described at the initial time *t*_0_, as the energy levels and their populations are defined. In a next step, the time-evolution of the populations must be considered and the resulting transverse magnetization at every time point has to be calculated. To this end, the full density matrix is required for every time point. The time-dependence of the density operator is described by the Liouville-von-Neumann equation, which can be easily derived from the definition of the density operator
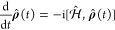
8which is solved by
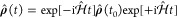
9

Here, the full Hamiltonian and the density matrix at *t*_0_ are needed as input parameters. Changes in the density matrix due to the microwave field are considered as part of the Hamiltonian.^39,60,62,69,70^ However, any further incoherent processes, like, for instance, relaxation, cannot be taken into account using this equation that emerged as a solution directly from the differential equation. The description of such processes requires a change to the density matrix operator itself.

This can be achieved by changing the mathematical space: In Hilbert space, operators act on functions, whereas in Liouville space (i.e., a space “above”) superoperators (marked with a double-hat) act on operators.^62^ Here, each matrix operator with the dimension *n* × *n* is written as a vector with *n*^2^ elements. These vectors are transformed by the superoperators, which are transformation matrices with the dimension *n*^2^ × *n*^2^. Hence, a superoperator that influences the density operator can be set up.

After transformation of the Liouville-von-Neumann equation into the Liouville space, incoherent dynamics can be taken into account by adding further superoperators. In doing so, the Liouville-von-Neumann equation turns into the stochastic Liouville equation^57,59,62^
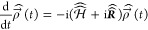
10which is solved by
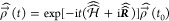
11where  is the time-independent dynamics superoperator which describes changes in the different population of the states and coherences of the system. Further superoperators, for instance for chemical reactions or for motion processes, could still be added. The Hamiltonian superoperator includes the commutator relation from eq 8.^60,62,67,70,71^

The dynamics superoperator is based on Redfield theory. This theory describes relaxation processes in open quantum systems by accounting for the interactions of the system with its environment. From these interactions, the elements of a relaxation superoperator are derived, which is then used in the stochastic Liouville equation.^72^ In teacups the elements of the relaxation superoperator are not derived from interactions with the environment, but are set directly. The construction of the dynamics superoperator, as performed in teacups, will be illustrated here for the two-state example system. As the operator dimension is 2 × 2, the superoperator has a dimension of 4 × 4 and takes the following general form
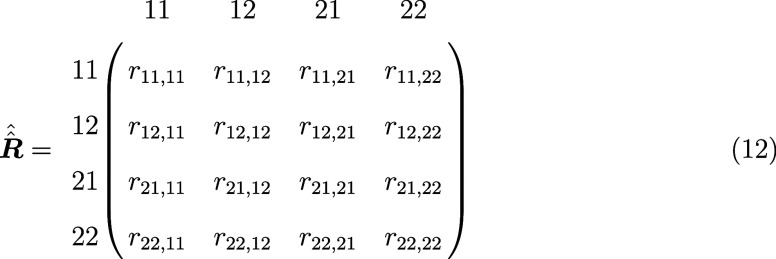
12where the indices *ij*, *kl* describe which elements *ij* and *kl* of the density matrix are affected by the rate constant *r*_*ij*,*kl*_. Density is transferred from the element *ij* to the element *kl*. Like this, modulations can be defined using the dynamics superoperator.^48,60,70,73^ The following rules apply:

- a positive *r* defines an increase of an element; a negative *r* a decrease
- *r* is always a rate constant that defines a proportionate change of the current value
- changes in populations are described by the elements with *i* = *j* and *k* = *l*
- changes in coherences are described by the elements with *i* ≠ *j* and *k* ≠ *l*
- the total population is only preserved if *∑*_*i*_*r*_*ii*,*kk*_ = 0
- population is transferred to states outside the spin system (e.g., decay to the ground state) for *∑*_*i*_*r*_*ii*,*kk*_ < 0, which can be accounted for by negative contributions in *r*_*ii*,*ii*_

In summary, the dynamics superoperator has the structure of the Redfield superoperator, but the (time-independent) rate constants are set directly instead of being calculated from interactions of the system with its environment. This approach is suitable for the analysis of photoexcited molecules, where the analysis of the rates can provide valuable insights into the internal dynamics of the spin system.

With the information given above, the density matrix can now be calculated for every time point. The only remaining step is then the calculation of the EPR signal itself. In transient continuous wave EPR, the detection is carried out perpendicular to the static magnetic field. Consequently, the spin operator along the *y*-axis is chosen as the detection operator. To obtain the EPR signal, the expectation value of the detection operator is calculated,^63,69^ which is defined as
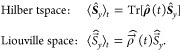
13

## 3 Results

In the following section, the specific features of teacups shall be outlined and explicit matrices and formulas will be given. teacups is an open-source Python package that can easily be implemented in self-written scripts. The script form gives maximal flexibility to the user; spin system and dynamics can be defined easily. A modular setup of the functions and a good documentation make it possible for users with some experience in Python to extend the program themselves by adding their own functions, e.g. for further spin systems, different initial populations or polarization transfer mechanisms. Details on the programming are given in the Supporting Information.

As explained in the theory section, the simulation input consists of three main parts: the spin system, the initial polarization, and the dynamic evolution, all together resulting in the simulated spectrum. For each part, teacups provides a selection of typical scenarios, that can be combined with each other. This makes the program easy to use, while further components can be added with knowledge of theory and programming. The available presets and their mathematical descriptions are presented in the following.

### 3.1 Basis and Basis Transformation

First a short comment on the use of different bases and the transformation between these bases shall be made: mathematical operations can be applied only if all matrices and vectors are defined in a vector space with the same basis. The basis of a vector space consists of the minimum number of linearly independent vectors. Each vector in the vector space can then be represented as a linear combination of these basis vectors.

For spectral simulations, it makes sense to use different bases depending on the situation. Examples are the eigenbasis of the Hamiltonian, in which it is diagonal, or the singlet–triplet basis for a radical pair, in which the initial occupation of the density matrix can be specified easily. Since the operators are partly set up in different bases, a basis transformation must be carried out in order to transfer all vectors and matrices into the same basis. The basis transformation matrix  transferring a matrix from the old basis *B* to the new basis *B*′ is set up by representing the vectors of the old basis as a linear combination of the vectors of the new basis. The basis transformation matrix is then applied to the vectors ***V*** and matrices ***M***(67,74)
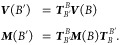
14

### 3.2 Powder Average

For the simulation of powder spectra of an anisotropic system, all orientations have to be considered equally. To do so, teacups calculates a powder average.

All tensors (after rotating them into a common initial frame) are rotated by a set of Euler angles using the rotation matrix as a basis transformation matrix. A rotation by ϕ about the *z*-axis is followed by a rotation by ϑ about the *y*-axis^75^
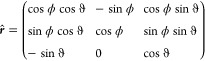
15

The set of angles consists of several pairs of ϕ and ϑ, describing points that are equally distributed on a unit sphere. For this purpose, a SOPHE^38^ grid and a Fibonacci grid^76^ are implemented. When rotating all tensors to a particular orientation, a spin system in this orientation is described. A powder sample consists of spin systems distributed across all possible orientations. By calculating spectra for a large number of orientations and summing them up with equal weights, a powder-averaged spectrum is obtained. In general, the more anisotropic the system, the more orientations have to be simulated to obtain a representative spectrum.^63^

As an alternative, teacups provides the possibility to calculate spectra only for a single orientation (or a small number of chosen orientations). This becomes useful when simulating trEPR spectra of oriented samples (e.g., for the study of chiral-induced spin selectivity (CISS)^77−79^).

### 3.3 Implemented Spin Systems

**Figure 3:**
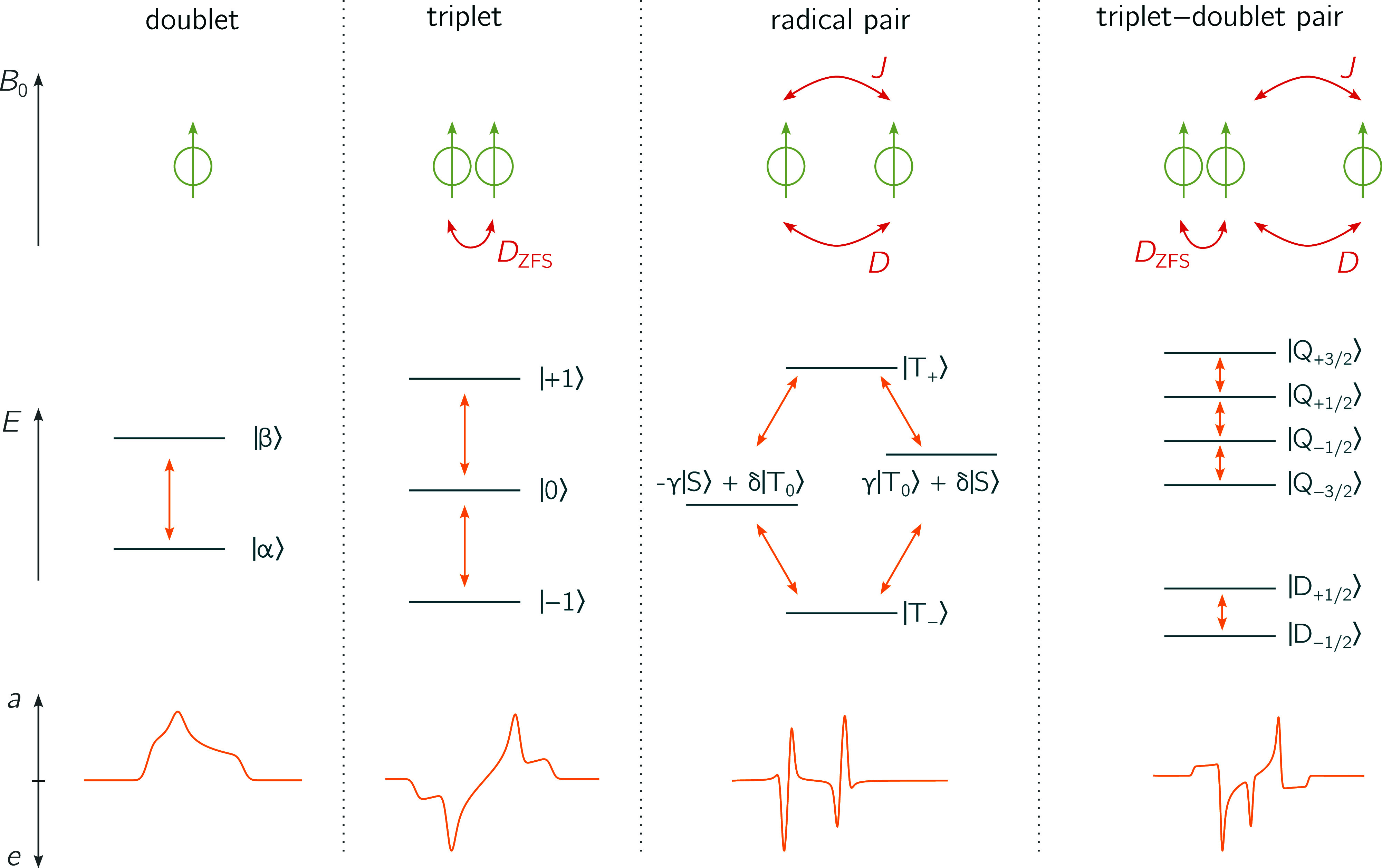
Overview of the implemented spin systems. The four spin systems that are currently implemented in teacups are shown. For each spin system, the involved spins and their interactions are sketched (top). Further, the eigenstates in a magnetic field and the allowed transitions between them are illustrated (center) together with a typical spin-polarized spectrum (bottom).

Predefined Hamiltonians for four spin systems (see Figure 3), that are often investigated using trEPR, are provided. These are a doublet (doub), a spin-correlated radical pair (rp), a triplet state (trip), and a triplet–doublet pair (tdp). Each species has its own set of magnetic interactions. The Hamiltonian matrices are shown in the Supporting Information.

The doublet consists of a single spin in a magnetic field. The spin quantum number is *s* = 1/2. In a magnetic field, two energy levels can be distinguished and one transition (resonant field) is expected. The Hamiltonian includes only the Zeeman interaction with the magnetic field
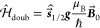
16

As a basis for all calculations, the two eigenfunctions |α⟩ and |β⟩ are chosen, which results in a diagonal Hamiltonian.^63^

The radical pair consists of two spins (with spin quantum numbers *s*_1,2_ = 1/2). This leads to a system of four spin states with four allowed transitions between them. Depending on the distance between the unpaired electron spins, exchange and/or dipolar interactions need to be taken into account. Both are considered in the Hamiltonian which takes the form^30^
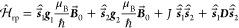
17

Instead of the product basis, where the functions are equal to the products of the radical eigenstates |α⟩ and |β⟩, the singlet–triplet basis is chosen
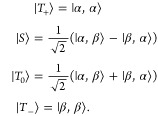
18

In this basis, the Hamiltonian has only few (two) and relatively small off-diagonal elements and can be assumed to be time-independent in the rotating frame.^30^

The triplet state consists of two spins in very close proximity. As a consequence, the exchange interaction, and therefore the energetic gap between the singlet and triplet states, becomes very large. The total system can be described by the three triplet functions, neglecting the singlet function. Hence, the total spin quantum number of a triplet is *S* = 1. Three triplet states exist and are connected by two allowed transitions. They are split by a dipolar interaction at zero field (zero-field-splitting, ZFS). The magnitude of the ZFS is defined by the two ZFS-parameters *D* and *E*. The corresponding Hamiltonian is given by
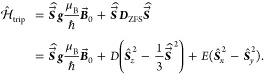
19When applying the secular approximation, only the *zz*-component of the **D**-tensor is taken into account,^63^ making the Hamiltonian time-independent in the rotating frame. As a basis for the triplet simulation, the eigenfunctions of the triplet state in high-field, |*T*_+_⟩, |*T*_0_⟩ and |*T*_–_⟩, are chosen.^37,66^

As the name implies, a triplet–doublet pair consists of a coupled triplet (*S* = 1) and a doublet (*s* = 1/2) state. This results in six eigenstates. Both the dipolar and exchange interactions need to be taken into account and are included in the Hamiltonian. The latter is similar to the Hamiltonian of the radical pair, except for the ZFS interaction that needs to be included for the triplet state
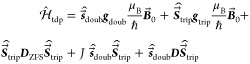
20

The magnitude of the exchange interaction defines the number of allowed transitions. For a strongly coupled pair, the quartet and doublet functions include four allowed transitions as shown in Figure 3. For moderately or weakly coupled systems (i.e., the magnitude of the spin–spin interactions is of the same order of (or smaller than) the Zeeman interaction), the energy differences between the eigenfunctions are small and additional transitions become allowed. As the basis, the product basis is chosen (products of the high-field triplet eigenfunctions and the doublet eigenfunctions). The secular approximation is applied for the dipolar coupling and the ZFS to make the Hamiltonian time-independent.^29,80−82^

### 3.4 Initial Polarization

Excited states can be generated by several different mechanisms leading to different types of initial polarizations (hereafter initial states). Some common initial states are already implemented and can be selected for the simulation.

The user can choose one out of five bases (explained in detail below) and set the initial populations of the states. The general internal procedure is then identical in each case: the density matrix is set up in the desired basis and then transformed into the basis of the Hamiltonian. For all implemented initial states, it is assumed that, initially, only longitudinal magnetization exists, i.e. coherences are neglected in the initial density matrices.

With a good knowledge of the theoretical concepts, it is possible for the user to provide a custom initial density matrix to the teacups simulation routine.

If the actual eigenstates of the system are to be populated, the initial state “eigen” has to be chosen. Here, the input populations are set as the diagonal elements of a matrix in a basis spanned by the systems eigenstates for every magnetic field point. Internally, the matrix is then transformed to the basis of the Hamiltonian
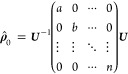
21

For this purpose, the basis transformation matrix ***U*** is used, that diagonalizes the Hamiltonian
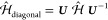
22

Most EPR spectra of spin-polarized triplet states can be described well when considering an initial population of the zero-field triplet eigenstates, for a recent review, see ref (66). In the program, the zero-field population can be selected by the user as the basis for a triplet state simulation by choosing the “zf”-initial state. Here, the populations that need to be given to the simulation routine are the populations of the three zero-field states of the triplet, |*T*_*X*_⟩, |*T*_*Y*_⟩ and |*T*_*Z*_⟩, in ascending order (with reference to their energies). The density matrix is transformed afterward to the high-field basis |*T*_+_⟩, |*T*_0_⟩ and |*T*_–_⟩ for each magnetic field point by basis transformation with the basis transformation matrix ***U***
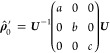
23

The matrix ***U*** is composed of the eigenvectors of the complete high-field Hamiltonian expressed in the *XYZ*-basis. Consequently, ***U*** can be determined by diagonalizing this high-field Hamiltonian.

To obtain , all off-diagonal elements, i.e. coherences, are set to zero after the basis transformation.^37,54,74^

For the coupled systems, namely the radical pair and the triplet–doublet pair, further initial states can be chosen. Radical pairs may be generated from a singlet or a triplet precursor. If “singlet” is chosen as the initial state, only the singlet state is populated and the population of all triplet states is set to zero. The initial density matrix is set up in the singlet–triplet basis according to
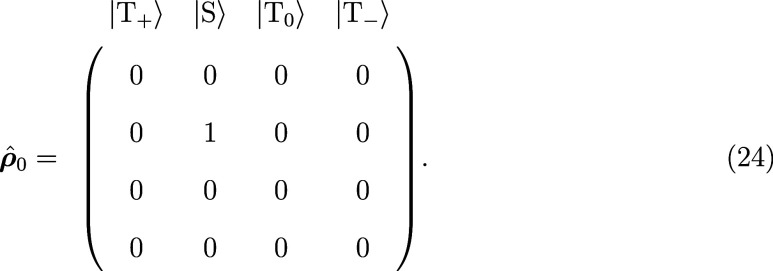
24

To simulate a radical pair with a triplet precursor, the three populations of the triplet sublevels have to be given. Here, it is possible to provide the high-field populations (“triplet-pnm”) or zero-field populations (“triplet-zf”). The density matrix of a triplet state is set up as explained above, populating the triplet levels of the radical pair density matrix.^54^

For the triplet–doublet pair, the density matrix is set up using two sets of populations: The population of the doublets and that of the triplets. Here again “triplet-pnm” or “triplet-zf” can be chosen, which defines the basis for the triplet. The doublet is set up in its eigenbasis states |α⟩ and |β⟩. As the Hamiltonian is set up in the product basis, the density matrix is also set up in the product basis of high-field triplet and doublet functions. To this end, the Kronecker product is computed^75^
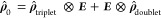
25

### 3.5 Implemented Relaxation Mechanisms

To simulate the time-evolution, relaxation models of different complexity can be chosen. First of all, the user needs to choose the mathematical space that is used for the calculations. This can either be “hilbert” or “liouville”.

If the Hilbert space is chosen, the advantage is that the operator dimensions are smaller. Therefore, calculations are much faster than in Liouville space. However, while coherent dynamics (like transient nutations^83^ and zero quantum coherences^35,84,85^) are simulated correctly, no dynamics superoperators can be applied. To simulate a phenomenological decay, the spectrum (*I*) is convoluted in teacups with an exponential decay of the form
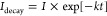
26characterized by the decay rate *k*.

If “liouville” is chosen, the operators are transformed into superoperators and vectors as described in the theory part. This squares their dimensions and slows down the computation, but dynamics superoperators can be included and incoherent dynamics can be simulated.^67^ Regarding the dynamics superoperator, different inputs are possible. The simplest way to simulate relaxation is by using the longitudinal relaxation time *T*_1_ and the transverse relaxation time *T*_2_. This describes a phenomenological decay of the signal but the model is more complex than a simple exponential decay and the decay times have a physical meaning: the longitudinal relaxation time drives the populations to equilibrium, while the transverse relaxation time describes the decay of the coherences. The relaxation superoperator is implemented using the relaxation times *T*_1_ and *T*_2_. The corresponding matrix can be found in the Supporting Information.^48,60,62,73^

Alternatively, for complete flexibility, the user can define their own dynamics superoperator (see the Supporting Information for limitations and advice). Since a specific model needs to be chosen, it is necessary to have some prior knowledge about the spin system and the feasibility of certain dynamic processes. Transitions between spin eigenstates and decay to the ground state can be initialized by defining the relevant matrix elements as described in the theory part.

### 3.6 Exemplary Workflow

**Figure 4:**
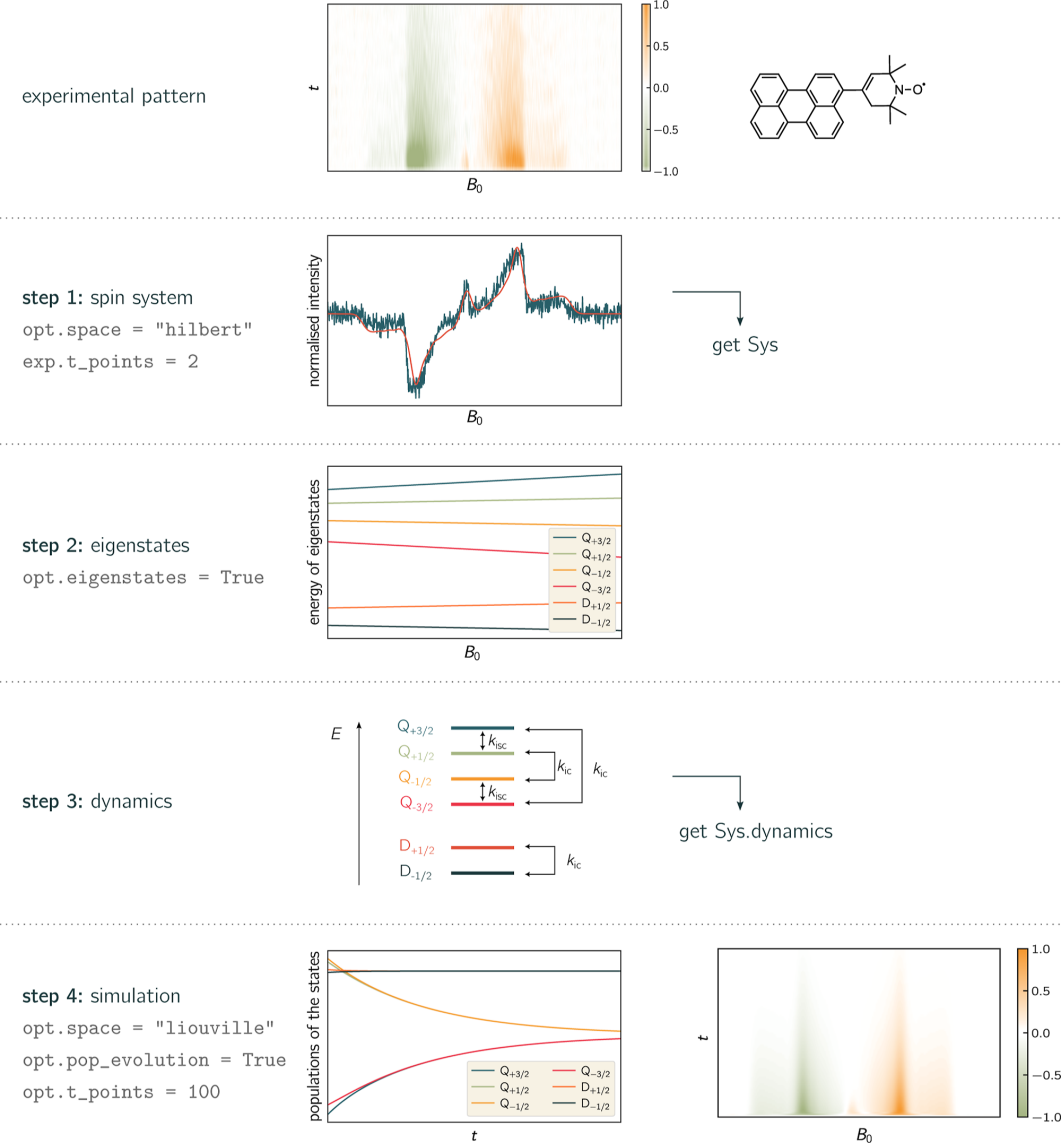
From an experimental spectrum to a teacups simulation. The simulation of a time-resolved EPR spectrum requires four steps which are illustrated here on the example of a photoexcited perylene–nitroxide molecule forming a triplet–doublet pair.^86^ The spin system parameters are determined by a quick Hilbert-space simulation of the EPR spectrum at early times after photoexcitation. The doublet and quartet eigenstates are plotted. Two rate constants, for intersystem crossing (isc) and internal conversion (ic), were chosen here to describe relaxation processes between the states. The whole spectrum and the evolution of the populations is finally plotted. Important simulation options are indicated in gray and the resulting plots are shown for each step. For a better illustration of the workflow, any ticks and tick labels were omitted in the plots of the experimental data. Experimental details and simulation parameters are provided in the Supporting Information (Sections S3, and S4).

In the following section, an example of how to use teacups is provided. The script used for the simulation (including the actual parameters used) can be found in the Supporting Information. The steps from an experimental time-resolved spectrum to the final 3D-simulation are explained and are visualized in Figure 4.

The transient EPR spectrum of a perylene–nitroxide conjugate, referred to as perylene-3-*e*TEMPO, is chosen as an example to illustrate the workflow of the program. It is a covalently bound, photogenerated triplet–doublet system, consisting of a stable radical and a chromophore. After photoexcitation at 435 nm, the chromophore triplet state is formed rapidly by a process referred to as radical-enhanced intersystem crossing.^29,86^ Since the chromophore triplet state and the radical doublet state are strongly coupled, four quartet states and two doublet states are formed. The experimental data set that is to be described is typical for a quartet state. The spectrum shows a broad multiplet polarization arising from transitions between the quartet state *Q*_±3/2_ ↔ *Q*_±1/2_ levels and a sharp net polarization in the center of the spectrum arising from transitions between the quartet state *Q*_+1/2_ ↔ *Q*_–1/2_ levels. When examining the experimental time-evolution of the signals, at least two different kinetic processes can be identified: the net polarization decays much faster than the multiplet polarization.^29^ Such a spectrum can be analyzed using teacups. To this end, the following workflow may be used:

1. characterization of the spin system: an EPR spectrum at early times after photoexcitation, which clearly shows all initial features, is chosen from the 3D data set. For a quick simulation, the time points are reduced to two and the Hilbert space is chosen. The resulting spectrum is then plotted. If required, the initial spin system parameters can be adjusted until a satisfactory agreement between experiment and simulation is reached. Further, an appropriate basis needs to be chosen, which may require a readjustment of the initial polarization. Now, the starting conditions are set.
2. Analysis of the eigenstate energies: now the eigenstates of the system can be verified. Upon request, teacups returns a plot of the energy levels of the eigenstates as a function of the magnetic field. In the case of our example, we can see here that the four quartet states are well separated from the two doublet states. From this plot, the energetic order of the states can be determined, which is needed to set up the matrix describing the dynamic processes.
3. Identification of plausible relaxation mechanisms: intersystem crossing (isc), describing transitions between states with different magnetic spin quantum numbers, and internal conversion (ic), describing transitions between states with the same magnetic spin quantum numbers, are plausible mechanisms leading to a decay of the signal and a redistribution of population. As a simple model, two rate constants *r*_isc_ and *r*_ic_ can be assumed. Now the respective states are connected with the rate constants in a dynamic matrix.
4. Calculation of the spectrum and the time-evolution of the eigenstate populations: the spectrum and the evolution of the populations can now be calculated. To this end, the Liouville space is chosen in combination with a sufficient number of time points. The rate constants are adjusted until the simulated signal decay fits the experimental data.

### 3.7 Further Simulation Examples

To illustrate some potential applications of teacups, an exemplary simulation is shown for each of the available spin systems. The simulation scripts including all simulation parameters are given in the Supporting Information.

**Figure 5:**
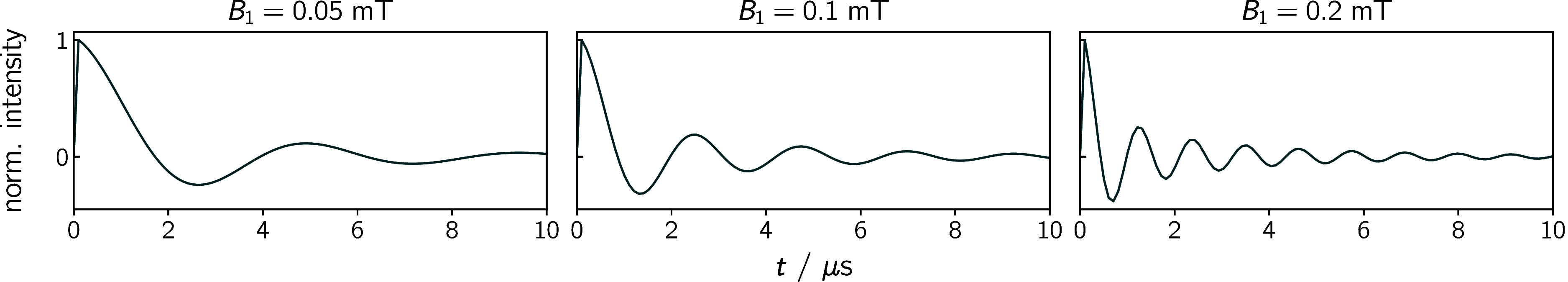
Hilbert space simulation for an isotropic doublet state. The time traces at the maximum peak intensity (*B*_0_ = 348.3 mT, ω_mw_ = 9.75 GHz) are shown for three different intensities of the microwave field *B*_1_ (as indicated). The observable transient nutations are proportional to *B*_1_. Further parameters used for the simulation are provided in the Supporting Information.

Starting with a simple doublet system (isotropic, no hyperfine couplings, *g* = 2.0), Figure 5 shows time traces at *B*_0_ = 348.3 mT, i.e. the maximum of the doublet peak at the chosen spectrometer frequency of 9.75 GHz, as a function of *B*_1_. The oscillations that can be observed in the simulation are transient nutations (Torrey oscillations)^83^ of the signal, which are proportional to *B*_1_. This behavior can be correctly reproduced in Hilbert space.

**Figure 6:**
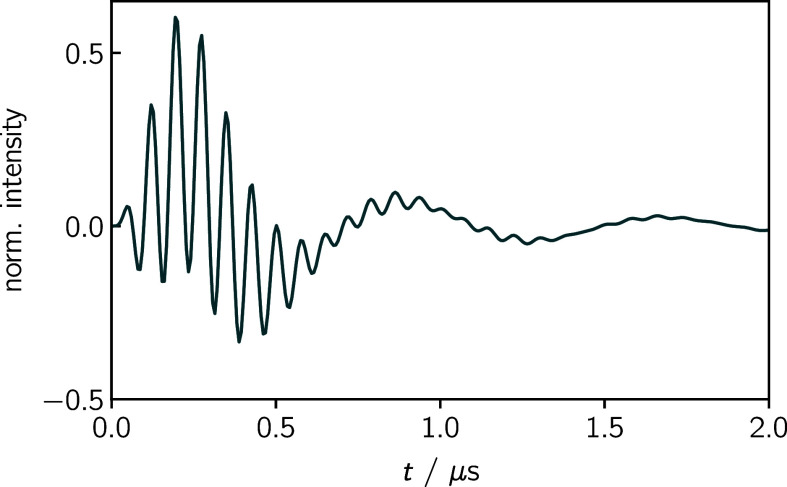
Hilbert space simulation for a radical pair. The parameters were taken from ref (48). The time trace is shown for *B*_0_ = 351.106 mT and ω_mw_ = 9.75 GHz. Apart from transient nutations, zero quantum coherence can be observed. Further parameters used for the simulation are provided in the Supporting Information.

Another Hilbert space simulation is shown for a radical pair in Figure 6. The time trace at the maximum peak intensity (*B*_0_ = 351.106 mT, ω_mw_ = 9.75 GHz) shows not only transient nutations but also zero quantum coherence, a phenomenon that is typical for radical pairs.^48,87^

**Figure 7:**
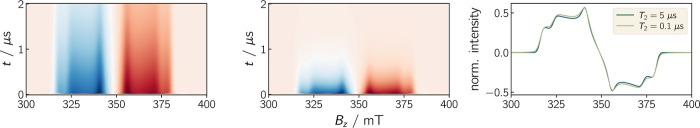
Influence of the phenomenological relaxation times*T*_1_ and*T*_2_ on a triplet spectrum. The spin system was adapted from ref (66). (Left) Spectrum simulated with *T*_1_ = *T*_2_ = 5 μs; (centre) *T*_1_ is shortened to 1 μs; (right) Comparison of triplet state spectra simulated for different *T*_2_ relaxation times (as indicated), with *T*_1_ = 5 μs. The spectra are shown at *t* = 1 μs after laser excitation. Further parameters used for the simulation are provided in the Supporting Information.

To illustrate the influence of the relaxation times *T*_1_ and *T*_2_, a Liouville space simulation of a triplet state spectrum, using a phenomenological relaxation superoperator, is shown in Figure 7. On the left-hand side, both relaxation times are equal: *T*_1_ = *T*_2_ = 5 μs. When *T*_1_ is shortened to 1 μs (*T*_2_ kept the same), the equilibration of the populations, and consequently the signal decay, is considerably faster (central panel in Figure 7). On the right-hand side, a comparison of two triplet state spectra at *t* = 1 μs after laser excitation is shown for *T*_1_ = 5 μs and two different *T*_2_ times (5 μs vs 0.1 μs). When *T*_2_ is shorter, the coherences decay faster and broadening effects become apparent.

**Figure 8:**
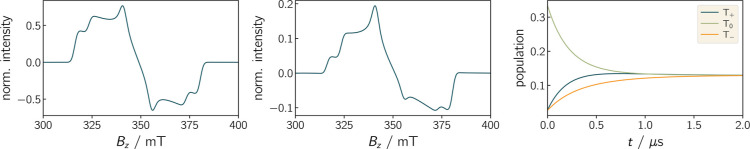
Triplet state simulations using a custom-defined relaxation matrix as shown in the text. Compared to the spectrum with equal relaxation rates for |*T*_±_⟩, the spectrum in the central panel is asymmetric. On the right-hand side, the evolution of the population of the eigenstates is shown. The spectra were simulated for *t* = 0.5 μs. Further parameters used for the simulation are provided in the Supporting Information.

By using a custom-defined relaxation matrix, more complicated dynamics can be simulated as shown in Figure 8. The left-most panel shows a triplet spectrum simulated using the same parameters as in Figure 7 (left panel). For differences in the relaxation times of the |T_±_⟩ states, an asymmetric transient spectrum is expected as described e.g. in ref (88). To simulate the spectrum shown in the central panel of Figure 8, the relaxation matrix was set to
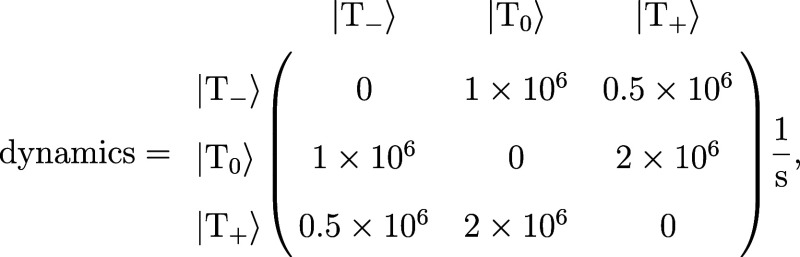
using different relaxation rates for the spin–lattice relaxation of |*T*_±_⟩ and neglecting the triplet state decay. The evolution of the population of the three triplet eigenstates is shown in the right-most panel.

**Figure 9:**
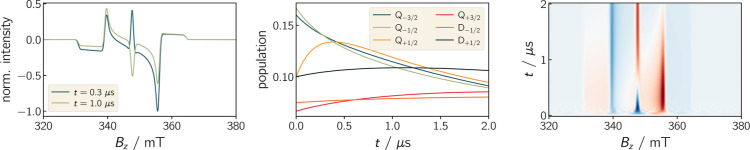
Simulation of the reverse-quartet mechanism. Different relaxation rates between the excited doublet states and the trip-quartet ±1/2 states lead to an inversion of the populations of the *Q* ± 1/2-states. This manifests in the spectrum in form of an inversion of the net polarization. Further parameters used for the simulation are provided in the Supporting Information.

Finally, a more complex example for a triplet–doublet pair is shown in Figure 9. Here a fictive spectrum is simulated illustrating the so-called reverse-quartet mechanism, which is frequently observed for strongly coupled pairs.^89^ Here, the excited *Q*_±1/2_ levels relax with different relaxation rates resulting in an inversion of the net polarization over time. From the example, it can be seen that this inversion of the central peak of the spectrum is caused by an inversion of the populations of the doublet states.

### 3.8 Specific Experimental Examples

In this section, we present the application of our simulation routine to experimental data, directly illustrating its usefulness for modeling and analyzing the time-dependent behavior of spin systems.

**Figure 10:**
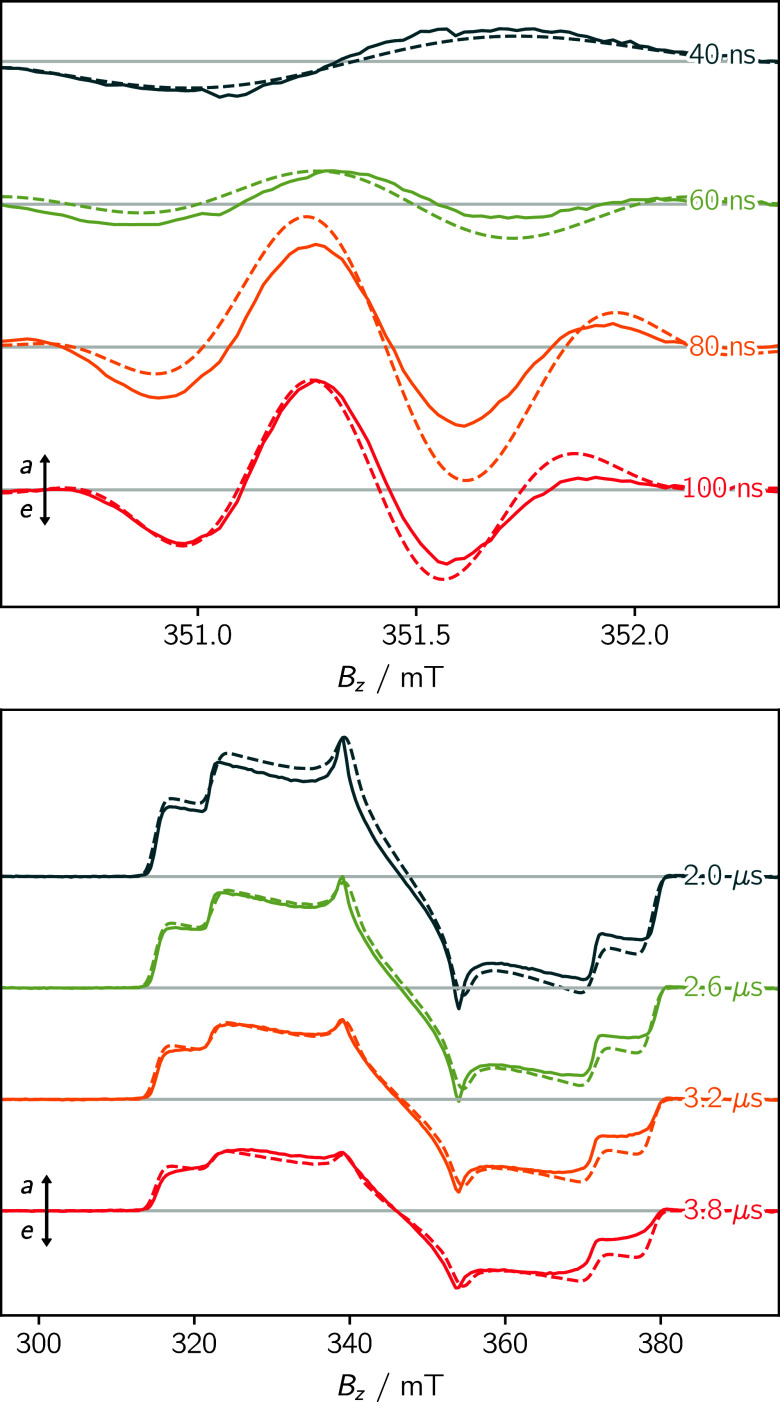
(Top) Fit of the time-resolved trEPR data of a photogenerated radical pair P_700_^+^A_1_^–^ in plant photosystem I reported in ref (48) (ω_mw_ = 9.8562 GHz, *B*_mw_ = 0.03 mT). (Bottom) Fit of the time-evolution of the trEPR data of the Zn-porphyrin monomer triplet state reported in ref (90). Positive and negative signals indicate absorptive and emissive transitions, respectively. The solid lines represent the experimental data, the dotted lines show the simulation using teacups. The simulation parameters are provided in the Supporting Information.

Figure 10top shows experimental data of a spin-correlated radical pair in plant photosystem I at four different time points within the first 100 ns after photoexcitation together with a teacups simulation that reproduces the time-resolved fit in ref (48). The good agreement between experiment and simulation demonstrates that the internal dynamics are reflected accurately. The simulation was performed in Hilbert-space and is influenced by all spin-system parameters (including the orientation between the tensors) and the experimental conditions, e.g. the microwave field strength. The full set of simulation parameters and the simulation script are provided in the Supporting Information.

As an example for the analysis of incoherent dynamics using teacups the time-resolved data of a zinc(II)-porphyrin triplet state from ref (90) were fitted as shown in Figure 10bottom. While the simulation of the static spectrum at *t* = 2 μs is shown in the reference, the time-evolution of the spectrum has not been analyzed previously. For the simulation using teacups, the spin system parameters were adapted from 90, while the dynamics-matrix was optimized. The chosen model was intentionally kept as simple as possible, including only transitions between the |*T*_±_⟩ and *T*_0_ states with different transition rates. By setting the simulation matrix to
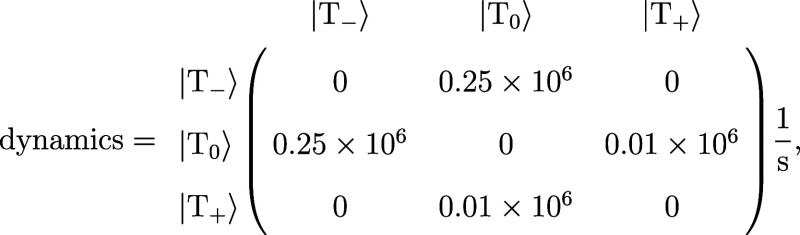


The decay of the amplitude and the increasing asymmetry of the spectrum could be reproduced satisfactorily.

## 4 Discussion & Conclusions

With teacups, a Python simulation routine for the simulation of time-resolved EPR spectra of spin-polarized species has been developed. It is possible to simulate powder spectra or spectra of oriented samples for doublets, triplets, spin-correlated radical pairs, and coupled doublet–triplet pairs. The spin system parameters can be chosen as well as the initial populations of the states. In addition, a flexible matrix can be defined that describes the dynamics of the system. After simulation of the spectrum, the evolution of the populations between the eigenstates can be compared with the experimental data. This makes it possible to analyze the effect of population transfer on the spectrum or to verify a plausible dynamic mechanism.

Teacups has a modular setup that guarantees maximal flexibility but has also some predefined models, that are easy to use. The modular setup allows the integration of further models. These can be further spin systems, like, for instance, triplet–triplet pairs or a doublet coupled to one or multiple nuclei,^91^ further dynamic processes (e.g., the reverse quartet mechanism),^92^ or further initial state matrices (e.g., with initially populated coherences).

In future, enhancing the programs efficiency should be a key focus. Currently, the time scale of a simulation is of the order of seconds to a few minutes depending on the spin system, the anisotropy (i.e., the required angular points/magnetic field points), and the length of the calculated time trace. The program can therefore be used to create individual simulations, but fitting is more difficult in a reasonable time. Details on initial efforts to improve efficiency are provided in the Supporting Information.

Additional simulation parameters could be included, such as hyperfine interactions. A further interesting feature to be added could be the transition between two different spin systems (e.g., accounting for the transition from the excited to the ground state; or the transition between different conformations). To this end, a full Hamiltonian consisting of the combination of both systems needs to be set up. Then, a superoperator matrix for the transition between all states can be defined. Functions of teacups for the build-up and propagation of the signal could readily be used for this purpose without changes.

Finally, we conclude that the presented simulation routine has great potential to develop into a flexible open source program for the simulation of time-resolved EPR spectra. This has been realized by providing a simple framework for the calculation of spin-polarized EPR spectra based on the density matrix formalism.

## Supporting Information Available

Comparison of static EPR spectra with EasySpin, explicit matrices for Hamiltonians and relaxation operators, information on the experimental data set in Figure 4, source code of exemplary simulations, details on the structure of the program (PDF)

## References

[ref1] Eills J.; Budker D.; Cavagnero S.; Chekmenev E. Y.; Elliott S. J.; Jannin S.; Lesage A.; Matysik J.; Meersmann T.; Prisner T.; et al. Spin hyperpolarization in modern magnetic resonance. Chem. Rev. 2023, 123, 1417–1551. 10.1021/acs.chemrev.2c00534. 36701528 PMC9951229

[ref2] Wolf S. A.; Awschalom D. D.; Buhrman R. A.; Daughton J. M.; von Molnár S.; Roukes M. L.; Chtchelkanova A. Y.; Treger D. M. Spintronics: A spin-based electronics vision for the future. Science 2001, 294, 1488–1495. 10.1126/science.1065389. 11711666

[ref3] Drummond B. H.; Aizawa N.; Zhang Y.; Myers W. K.; Xiong Y.; Cooper M. W.; Barlow S.; Gu Q.; Weiss L. R.; Gillett A. J.; et al. Electron spin resonance resolves intermediate triplet states in delayed fluorescence. Nat. Commun. 2021, 12, 4532 10.1038/s41467-021-24612-9. 34312394 PMC8313702

[ref4] Levanon H.; Norris J. R. The photoexcited triplet state and photosynthesis. Chem. Rev. 1978, 78, 185–198. 10.1021/cr60313a001.

[ref5] Budil D. E.; Thurnauer M. C. The chlorophyll triplet state as a probe of structure and function in photosynthesis. Biochim. Biophys. Acta 1991, 1057, 1–41. 10.1016/S0005-2728(05)80081-7. 1849002

[ref6] Rao A.; Chow P. C. Y.; Gélinas S.; Schlenker C. W.; Li C.-Z.; Yip H.-L.; Jen A. K.-Y.; Ginger D. S.; Friend R. H. The role of spin in the kinetic control of recombination in organic photovoltaics. Nature 2013, 500, 435–439. 10.1038/nature12339. 23925118

[ref7] Niklas J.; Poluektov O. G. Charge transfer processes in OPV materials as revealed by EPR spectroscopy. Adv. Energy Mater. 2017, 7, 1602226 10.1002/aenm.201602226.

[ref8] Di Valentin M.; Albertini M.; Zurlo E.; Gobbo M.; Carbonera D. Porphyrin triplet state as a potential spin label for nanometer distance measurements by PELDOR spectroscopy. J. Am. Chem. Soc. 2014, 136, 6582–6585. 10.1021/ja502615n. 24735449

[ref9] Hintze C.; Bücker D.; Domingo Köhler S.; Jeschke G.; Drescher M. Laser-induced magnetic dipole spectroscopy. J. Phys. Chem. Lett. 2016, 7, 2204–2209. 10.1021/acs.jpclett.6b00765. 27163749

[ref10] Bieber A.; Bücker D.; Drescher M. Light-induced dipolar spectroscopy - A quantitative comparison between LiDEER and LaserIMD. J. Magn. Reson. 2018, 296, 29–35. 10.1016/j.jmr.2018.08.006. 30199790

[ref11] Dal Farra M. G.; Richert S.; Martin C.; Larminie C.; Gobbo M.; Bergantino E.; Timmel C. R.; Bowen A. M.; Di Valentin M. Light-induced pulsed EPR dipolar spectroscopy on a paradigmatic hemeprotein. ChemPhysChem 2019, 20, 931–935. 10.1002/cphc.201900139. 30817078 PMC6618045

[ref12] Hore P. J.; Riley D. J.; Semlyen J. J.; Zwanenburg G.; Hoff A. J. Analysis of anisotropic electron spin polarization in the photosynthetic bacterium *Rhodospirillum Rubrum*.: Evidence that the sign of the exchange interaction in the primary radical pair is positive. Biochim. Biophys. Acta 1993, 1141, 221–230. 10.1016/0005-2728(93)90046-I.

[ref13] Hasharoni K.; Levanon H.; Greenfield S. R.; Gosztola D. J.; Svec W. A.; Wasielewski M. R. Radical pair and triplet state dynamics of a photosynthetic reaction-center model embedded in isotropic media and liquid crystals. J. Am. Chem. Soc. 1996, 118, 10228–10235. 10.1021/ja961919e.

[ref14] Lubitz W.; Lendzian F.; Bittl R. Radicals, radical pairs and triplet states in photosynthesis. Acc. Chem. Res. 2002, 35, 313–320. 10.1021/ar000084g. 12020169

[ref15] Bittl R.; Weber S. Transient radical pairs studied by time-resolved EPR. Biochim. Biophys. Acta 2005, 1707, 117–126. 10.1016/j.bbabio.2004.03.012. 15721610

[ref16] Weber S. Light-driven enzymatic catalysis of DNA repair: A review of recent biophysical studies on photolyase. Biochim. Biophys. Acta 2005, 1707, 1–23. 10.1016/j.bbabio.2004.02.010. 15721603

[ref17] Biskup T.; Schleicher E.; Okafuji A.; Link G.; Hitomi K.; Getzoff E.; Weber S. Direct observation of a photoinduced radical pair in a cryptochrome blue-light photoreceptor. Angew. Chem., Int. Ed. 2009, 48, 404–407. 10.1002/anie.200803102. PMC4329312 19058271

[ref18] Xu J.; Jarocha L. E.; Zollitsch T.; Konowalczyk M.; Henbest K. B.; Richert S.; Golesworthy M. J.; Schmidt J.; Déjean V.; Sowood D. J. C.; et al. Magnetic sensitivity of cryptochrome 4 from a migratory songbird. Nature 2021, 594, 535–540. 10.1038/s41586-021-03618-9. 34163056

[ref19] Hochstoeger T.; Al Said T.; Maestre D.; Walter F.; Vilceanu A.; Pedron M.; Cushion T. D.; Snider W.; Nimpf S.; Nordmann G. C.; et al. The biophysical, molecular, and anatomical landscape of pigeon CRY4: A candidate light-based quantal magnetosensor. Sci. Adv. 2020, 6, eabb9110 10.1126/sciadv.abb9110. 32851187 PMC7423367

[ref20] van der Est A.; Prisner T.; Bittl R.; Fromme P.; Lubitz W.; Möbius K.; Stehlik D. Time-resolved X-, K-, and W-band EPR of the radical pair state P^+^_700_ A^–^_1_of photosystem I in comparison with P^+^_865_ Q^–^_A_ in bacterial reaction centers. J. Phys. Chem. B 1997, 101, 1437–1443. 10.1021/jp9622086.

[ref21] Bittl R.; Zech S. G. Pulsed EPR spectroscopy on short-lived intermediates in photosystem I. Biochim. Biophys. Acta 2001, 1507, 194–211. 10.1016/S0005-2728(01)00210-9. 11687215

[ref22] Weber S.; Biskup T.; Okafuji A.; Marino A. R.; Berthold T.; Link G.; Hitomi K.; Getzoff E. D.; Schleicher E.; Norris J. R. Origin of light-induced spin-correlated radical pairs in cryptochrome. J. Phys. Chem. B 2010, 114, 14745–14754. 10.1021/jp103401u. 20684534 PMC4329313

[ref23] Walker B. J.; Musser A. J.; Beljonne D.; Friend R. H. Singlet exciton fission in solution. Nat. Chem. 2013, 5, 1019–1024. 10.1038/nchem.1801. 24256865

[ref24] Weiss L. R.; Bayliss S. L.; Kraffert F.; Thorley K. J.; Anthony J. E.; Bittl R.; Friend R. H.; Rao A.; Greenham N. C.; Behrends J. Strongly exchange-coupled triplet pairs in an organic semiconductor. Nat. Phys. 2017, 13, 176–181. 10.1038/nphys3908.

[ref25] Tayebjee M. J. Y.; Sanders S. N.; Kumarasamy E.; Campos L. M.; Sfeir M. Y.; McCamey D. R. Quintet multiexciton dynamics in singlet fission. Nat. Phys. 2017, 13, 182–188. 10.1038/nphys3909.

[ref26] Bae Y. J.; Zhao X.; Kryzaniak M. D.; Nagashima H.; Strzalka J.; Zhang Q.; Wasielewski M. R. Spin dynamics of quintet and triplet states resulting from singlet fission in oriented terrylenediimide and quaterrylenediimide films. J. Phys. Chem. C 2020, 124, 9822–9833. 10.1021/acs.jpcc.0c03189.

[ref27] Mayländer M.; Chen S.; Lorenzo E. R.; Wasielewski M. R.; Richert S. Exploring photogenerated molecular quartet states as spin qubits and qudits. J. Am. Chem. Soc. 2021, 143, 7050–7058. 10.1021/jacs.1c01620. 33929834

[ref28] Wasielewski M. R.; Forbes M. D. E.; Frank N. L.; Kowalski K.; Scholes G. D.; Yuen-Zhou J.; Baldo M. A.; Freedman D. E.; Goldsmith R. H.; Goodson T. III; et al. Exploiting chemistry and molecular systems for quantum information science. Nat. Rev. Chem 2020, 4, 490–504. 10.1038/s41570-020-0200-5. 37127960

[ref29] Quintes T.; Mayländer M.; Richert S. Properties and applications of photoexcited chromophore-radical systems. Nat. Rev. Chem 2023, 7, 75–90. 10.1038/s41570-022-00453-y. 37117913

[ref30] Harvey S. M.; Wasielewski M. R. Photogenerated spin-correlated radical pairs: From photosynthetic energy transduction to quantum information science. J. Am. Chem. Soc. 2021, 143, 15508–15529. 10.1021/jacs.1c07706. 34533930

[ref31] Mayländer M.; Thielert P.; Quintes T.; Vargas Jentzsch A.; Richert S. Room temperature electron spin coherence in photogenerated molecular spin qubit candidates. J. Am. Chem. Soc. 2023, 145, 14064–14069. 10.1021/jacs.3c04021. 37337625

[ref32] Khariushin I. V.; Thielert P.; Zöllner E.; Mayländer M.; Quintes T.; Richert S.; Vargas Jentzsch A. Supramolecular dyads as photogenerated qubit candidates. Nat. Chem. 2025, 10.1038/s41557-024-01716-5. 39870812

[ref33] Weber S. Transient EPR. eMagRes 2017, 6, 255–270. 10.1002/9780470034590.emrstm1509.

[ref34] Stehlik D.; Bock C. H.; Petersen J. Anisotropic electron spin polarization of correlated spin pairs in photosynthetic reaction centers. J. Phys. Chem. 1989, 93, 1612–1619. 10.1021/j100341a084.

[ref35] Kothe G.; Weber S.; Bittl R.; Ohmes E.; Thurnauer M. C.; Norris J. R. Transient EPR of light-induced radical pairs in plant photosystem I: observation of quantum beats. Chem. Phys. Lett. 1991, 186, 474–480. 10.1016/0009-2614(91)90454-H.

[ref36] Liang Z.; Freed J. H. An assessment of the applicability of multifrequency ESR to study the complex dynamics of biomolecules. J. Phys. Chem. B 1999, 103, 6384–6396. 10.1021/jp9907746.

[ref37] Tait C. E.; Krzyaniak M. D.; Stoll S. Computational tools for the simulation and analysis of spin-polarized EPR spectra. J. Magn. Reson. 2023, 349, 107410 10.1016/j.jmr.2023.107410. 36870248

[ref38] Stoll S.; Schweiger A. EasySpin, a comprehensive software package for spectral simulation and analysis in EPR. J. Magn. Reson. 2006, 178, 42–55. 10.1016/j.jmr.2005.08.013. 16188474

[ref39] Pribitzer S.; Doll A.; Jeschke G. SPIDYAN, a MATLAB library for simulating pulse EPR experiments with arbitrary waveform excitation. J. Magn. Reson. 2016, 263, 45–54. 10.1016/j.jmr.2015.12.014. 26773526

[ref40] Hogben H.; Krzystyniak M.; Charnock G.; Hore P.; Kuprov I. Spinach – A software library for simulation of spin dynamics in large spin systems. J. Magn. Reson. 2011, 208 (2), 179–194. 10.1016/j.jmr.2010.11.008. 21169043

[ref41] McLauchlan K. A.; Sealy R. C.; Wittmann J. M. Electron spin relaxation in polarized secondary radicals: Part 1. Theory. Mol. Phys. 1978, 35, 51–63. 10.1080/00268977800100041.

[ref42] Hore P. J.; Joslin C. G.; McLauchlan K. A. The role of chemically-induced dynamic electron polarization (CIDEP) in chemistry. Chem. Soc. Rev. 1979, 8, 29 10.1039/cs9790800029.

[ref43] Gonen O.; Levanon H. Time-resolved EPR spectroscopy of electron spin polarized ZnTPP triplets oriented in a liquid crystal. J. Phys. Chem. 1985, 89, 1637–1643. 10.1021/j100255a017.

[ref44] McLauchlan K. A.; Ritchie A. J. D. A flash-photolysis electron spin resonance study of radicals formed from carboxylic acids; exchange effects in spin-polarized radicals. Mol. Phys. 1985, 56, 141–159. 10.1080/00268978500102231.

[ref45] Hore P. J.; Watson E.; Pedersen J. B.; Hoff A. J. Line-shape analysis of polarized electron paramagnetic resonance spectra of the primary reactants of bacterial photosynthesis. Biochim. Biophys. Acta 1986, 849, 70–76. 10.1016/0005-2728(86)90097-6.

[ref46] Schneider D. J.; Freed J. H. Advances in Chemical PhysicsChapter Spin relaxation and motional dynamics; John Wiley & Sons, Ltd, 1989; pp 387–527.

[ref47] Corvaja C.; Franco L.; Pasimeni L.; Toffoletti A. Time resolved EPR of triplet excitons in phenazine-TCNQ charge transfer crystal. Appl. Magn. Reson. 1992, 3, 797–813. 10.1007/BF03260112.

[ref48] Kothe G.; Weber S.; Ohmes E.; Thurnauer M. C.; Norris J. R. Transient EPR of light-induced spin-correlated radical pairs: Manifestation of zero quantum coherence. J. Phys. Chem. 1994, 98, 2706–2712. 10.1021/j100061a031.

[ref49] Tarasov V. F.; Bagranskaya E. G.; Shkrob I. A.; Avdievich N. I.; Ghatlia N. D.; Lukzen N. N.; Turro N. J.; Sagdeev R. Z. Examination of the exchange interaction through micellar size. 3. Stimulated nuclear polarization and time resolved electron spin resonance spectra from the photolysis of methyldeoxybenzoin in alkyl sulfate micelles of different sizes. J. Am. Chem. Soc. 1995, 117, 110–118. 10.1021/ja00106a014.

[ref50] Elger G.; Fuhs M.; Müller P.; Gersdorff J.; Wiehe A.; Kurreck H.; Möbius K. Time-resolved EPR studies of photoinduced electron transfer reactions in photosynthetic model porphyrin quinone triads. Mol. Phys. 1998, 95, 1309–1323.

[ref51] Conti F.; Corvaja C.; Gattazzo C.; Toffoletti A.; Bergo P.; Maggini M.; Scorrano G.; Prato M. Time-resolved EPR investigation of intramolecular photoinduced electron transfer in spin-labeled fullerene/ferrocene dyads. Phys. Chem. Chem. Phys. 2001, 3, 3526–3531. 10.1039/b104482f.

[ref52] Salikhov K. M.; Pushkar Y. N.; Golbeck J. H.; Stehlik D. Interpretation of multifrequency transient EPR spectra of the P^+^_700_ A_0_Q^–^_K_state in photosystem I complexes with a sequential correlated radical pair model: Wild type versus A_0_ mutants. Appl. Magn. Reson. 2003, 24, 467–482. 10.1007/BF03166949.

[ref53] Mi Q.; Ratner M. A.; Wasielewski M. R. Time-resolved EPR spectra of spin-correlated radical pairs: Spectral and kinetic modulation resulting from electron-nuclear hyperfine interactions. J. Phys. Chem. A 2010, 114, 162–171. 10.1021/jp907476q. 19994848

[ref54] Kobori Y.; Fuki M.; Murai H. Electron spin polarization transfer to the charge-separated state from locally excited triplet configuration: Theory and its application to characterization of geometry and electronic coupling in the electron donor-acceptor system. J. Phys. Chem. B 2010, 114, 14621–14630. 10.1021/jp102330a. 20509645

[ref55] Bagryansky V. A.; Borovkov V. I.; Molin Y. N. The time-resolved magnetic field effect in the spin-dependent recombination of immobile radical ion pairs. Appl. Magn. Reson. 2022, 53, 581–593. 10.1007/s00723-021-01368-5.

[ref56] Kubo R. Stochastic Liouville equations. J. Math. Phys. 1963, 4, 174–183. 10.1063/1.1703941.

[ref57] Kubo R. In Advances in Chemical Physics: Stochastic Processes in Chemical Physics; Shuler K. E., Ed.; Chapter Chapter X: A Stochastic Theory of Line Shape; John Wiley & Sons, Inc., 1969, pp 101–127.

[ref58] Freed J. H. Electron spin resonance. Annu. Rev. Phys. Chem. 1972, 23, 265–310. 10.1146/annurev.pc.23.100172.001405. 11031296

[ref59] Vega A. J.; Fiat D. The stochastic liouville equation and the approach to thermal equilibrium. Pure Appl. Chem. 1974, 40, 181–192. 10.1351/pac197440010181.

[ref60] Feintuch A.; Vega S. Spin dynamics. eMagRes 2017, 6, 427–452. 10.1002/9780470034590.emrstm1506.

[ref61] Atherton N. M. Principles of Electron Spin Resonance, 1st ed.; Ellis Horwood Limited: Chichester, 1993.

[ref62] Ernst R. R.; Bodenhausen G.; Wokaun A. Principles of Nuclear Magnetic Resonance in One and Two Dimensions, 1st ed.; Clarendon Press: Oxford, 1987.

[ref63] Levitt M. H. Spin dynamics. In Basics of Nuclear Magnetic Resonance, 2nd ed.; John Wiley & Sons Ltd: Chichester, 2015.

[ref64] Weil J. A.; Bolton J. R. Electron Paramagnetic Resonance, 2nd ed.; John Wiley & Sons Inc.: Hoboken, 2007.

[ref65] McInnes E. J. L.; Collison D. EPR interactions - Coupled spins. eMagRes 2016, 5, 1445–1458. 10.1002/9780470034590.emrstm1502.

[ref66] Richert S.; Tait C. E.; Timmel C. R. Delocalisation of photoexcited triplet states probed by transient EPR and hyperfine spectroscopy. J. Magn. Reson. 2017, 280, 103–116. 10.1016/j.jmr.2017.01.005. 28579096

[ref67] Gyamfi J. A. Fundamentals of quantum mechanics in Liouville space. Eur. J. Phys. 2020, 41, 063002 10.1088/1361-6404/ab9fdd.

[ref68] Blum K. Density matrix theory and applications. In Springer Series on Atomic, Optical and Plasma Physics, 3rd ed.; Springer-Verlag: Berlin: Heidelberg, 2012; Vol. 64.

[ref69] Nitzan A. Chemical dynamics in condensed phases. Relaxation, Transfer, And Reactions in Condensed Molecular Systems, 1st ed.; Oxford University Press Inc.: New York, 2006.

[ref70] Schweiger A.; Jeschke G. Principles of Pulse Electron Paramagnetic Resonance, 1st ed.; Oxford University Press: Oxford, 2001.

[ref71] Borbat P. P.; Freed J. H. Dipolar spectroscopy - Single-resonance methods. eMagRes 2017, 6, 465–494. 10.1002/9780470034590.emrstm1519.

[ref72] Redfield A. In Advances in magnetic Resonance: The theory of relaxation processes; Waugh J. S., Ed.; Academic Press, 1965; Vol. 1, pp 1–32.

[ref73] Tarasov V. F.; Saiful I. S. M.; Iwasaki Y.; Ohba Y.; Savitsky A.; Möbius K.; Yamauchi S. Electron spin polarization in an excited triplet-radical pair system: Generation and decay of the state. Appl. Magn. Reson. 2006, 30, 619–636. 10.1007/BF03166222.

[ref74] Anton H.; Rorres C. Elementary Linear Algebra, 11th ed.; John Wiley & Sons Inc.: Hoboken, 2014.

[ref75] Thompson W. J. Angular momentum. In An illustrated Guide to Rotational Symmetries for Physical Systems, 1st ed.; Wiley VCH: Weinheim, 2004.

[ref76] Zadrozny J. M.; Niklas J.; Poluektov O. G.; Freedman D. E. Multiple quantum coherences from hyperfine transitions in a vanadium(IV) complex. J. Am. Chem. Soc. 2014, 136, 15841–15844. 10.1021/ja507846k. 25340518

[ref77] Luo J.; Hore P. J. Chiral-induced spin selectivity in the formation and recombination of radical pairs: cryptochrome magnetoreception and EPR detection. New J. Phys. 2021, 23, 043032 10.1088/1367-2630/abed0b.

[ref78] Fay T. P. Chirality-induced spin coherence in electron transfer reactions. J. Phys. Chem. Lett. 2021, 12, 1407–1412. 10.1021/acs.jpclett.1c00009. 33513302

[ref79] Eckvahl H. J.; Tcyrulnikov N. A.; Chiesa A.; Bradley J. M.; Young R. M.; Carretta S.; Krzyaniak M. D.; Wasielewski M. R. Direct observation of chirality-induced spin selectivity in electron donor–acceptor molecules. Science 2023, 382, 197–201. 10.1126/science.adj5328. 37824648

[ref80] Kandrashkin Y. E.; van der Est A. Stimulated electron spin polarization in strongly coupled triplet-doublet spin pairs. Appl. Magn. Reson. 2011, 40, 189–204. 10.1007/s00723-011-0194-8.

[ref81] Kandrashkin Y. E.; van der Est A. The triplet mechanism of electron spin polarization in moderately coupled triplet-doublet rigid complexes as a source of the enhanced + 1/2 ↔ −1/2 transitions. J. Chem. Phys. 2019, 151, 184301 10.1063/1.5127762. 31731838

[ref82] Kollmar C.; Sixl H. Theory of a coupled doublet-triplet system. Mol. Phys. 1982, 45, 1199–1208. 10.1080/00268978200100921.

[ref83] Torrey H. C. Transient nutations in nuclear magnetic resonance. Phys. Rev. 1949, 76, 1059–1068. 10.1103/PhysRev.76.1059.

[ref84] Bittl R.; Kothe G. Transient EPR of radical pairs in photosynthetic reaction centers: prediction of quantum beats. Chem. Phys. Lett. 1991, 177, 547–553. 10.1016/0009-2614(91)90082-K.

[ref85] Salikhov K. M.; Bock C. H.; Stehlik D. Time development of electron spin polarization in magnetically coupled, spin correlated radical pairs. Appl. Magn. Reson. 1990, 1, 195–211. 10.1007/BF03166155.

[ref86] Thielert P.; El Bitar Nehme M.; Mayländer M.; Franz M.; Zimmermann S. L.; Fisch F.; Gilch P.; Vargas Jentzsch A.; Rickhaus M.; Richert S. Influence of the substitution position on spin communication in photoexcited perylene-nitroxide dyads. Chem. Sci. 2024, 15, 7515–7523. 10.1039/D4SC00328D. 38784753 PMC11110163

[ref87] Zwanenburg G.; Hore P. J. EPR of spin-correlated radical pairs. Analytical treatment of selective excitation including zero-quantum coherence. Chem. Phys. Lett. 1993, 203, 65–74. 10.1016/0009-2614(93)89312-6.

[ref88] Hintze C.; Steiner U. E.; Drescher M. Photoexcited triplet state kinetics studied by electron paramagnetic resonance spectroscopy. ChemPhysChem 2017, 18, 6–16. 10.1002/cphc.201600868. 27791329

[ref89] Giacobbe E. M.; Mi Q.; Colvin M. T.; Cohen B.; Ramanan C.; Scott A. M.; Yeganeh S.; Marks T. J.; Ratner M. A.; Wasielewski M. R. Ultrafast intersystem crossing and spin dynamics of photoexcited perylene-3,4:9,10-bis(dicarboximide) covalently linked to a nitroxide radical at fixed distances. J. Am. Chem. Soc. 2009, 131, 3700–3712. 10.1021/ja808924f. 19231866

[ref90] Tait C. E.; Neuhaus P.; Anderson H. L.; Timmel C. R. Triplet state delocalization in a conjugated porphyrin dimer probed by transient electron paramagnetic resonance techniques. J. Am. Chem. Soc. 2015, 137, 6670–6679. 10.1021/jacs.5b03249. 25914154 PMC4569061

[ref91] Kawai A.; Shibuya K. Electron spin dynamics in a pair interaction between radical and electronically-excited molecule as studied by a time-resolved ESR method. J. Photochem. Photobiol., C 2006, 7, 89–103. 10.1016/j.jphotochemrev.2006.06.001.

[ref92] Rozenshtein V.; Berg A.; Stavitski E.; Levanon H.; Franco L.; Corvaja C. Electron spin polarization of functionalized fullerenes. Reversed quartet mechanism. J. Phys. Chem. A 2005, 109, 11144–11154. 10.1021/jp0540104. 16331897

