# Supplementary material for: Teacups, a Python Package for the Simulation of Time-Resolved EPR Spectra of Spin-Polarized Multi-Spin Systems
Source: J Phys Chem A. 2025 Mar 28;129(14):3375–88. doi: 10.1021/acs.jpca.5c01512 (PMC11995384; doi:10.1021/acs.jpca.5c01512)
Supplement: Supplementary file 1 — jp5c01512_si_001.pdf [file jp5c01512_si_001.pdf]

Supporting Information (SI)

Teacups, a Python Package for the Simulation of Time-Resolved EPR Spectra of Spin-Polarized Multi-Spin Systems

Theresia Quintes, Stefan Weber, Sabine Richert*

Institute of Physical Chemistry, University of Freiburg, Albertstraße 21, 79104 Freiburg, Germany
sabine.richert@physchem.uni-freiburg.de

Contents

1 Comparison of static, anisotropic spectra with EasySpin S1

2 Explicit matrices S1

2.1 Hamiltonians . . . S1

2.2 Relaxation . . . S2

3 Experimental details for Figure 4 S3

4 Simulation parameters S5

5 Source code of the simulations S6

6 Programming details S13

6.1 Specific simulation modules . . . S13

6.2 General modules . . . S13

6.3 Structuring modules . . . S14

7 Code optimization S14

1 Comparison of static, anisotropic spectra with EasySpin

In order to verify whether the secular approximation used in our implementation leads to satisfactory results, two spectrally broad, anisotropic spin systems were simulated with teacups and the results compared with EasySpin simulations. The perfect agreement (cf. Figure S1) demonstrates the validity of the approach.

Table S1: Parameters used in the simulations.

Triplet	
g	2.003
D	700 MHz
E	−100 MHz
precursor	"zf"
population	[1, 0, 0]
**Triplet–doublet pair**	
g_r	2.0059
g_t	2.003
D_{ZFS}	500 MHz
E_{ZFS}	−50 MHz
J	−400000 MHz
precursor	"triplet-zf"
population	[0.2, 0.2, 1, 0, 0]

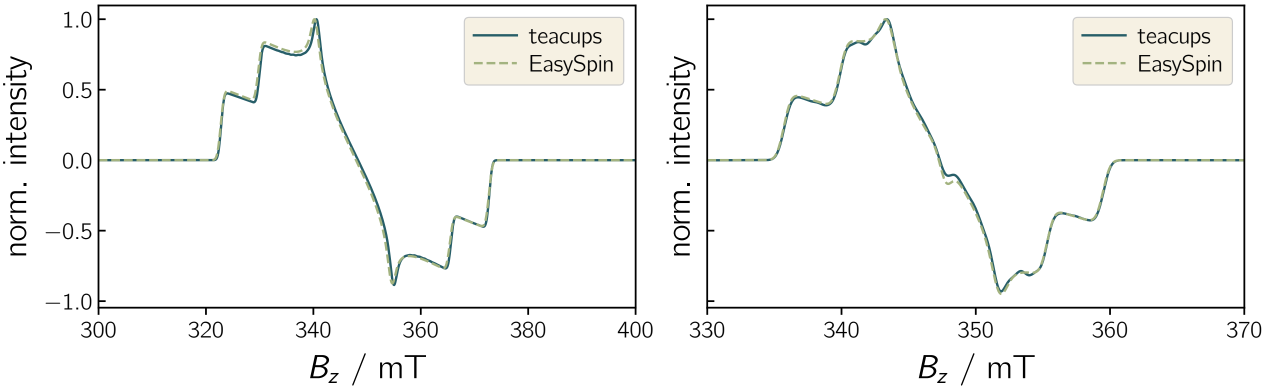

Figure S1: Comparison of static EPR spectra simulated with teacups (using the secular approximation) and EasySpin (no secular approximation). *Left*: Triplet state spectrum, *Right*: Triplet–doublet pair. The simulation parameters are provided in the table above.

2 Explicit matrices

2.1 Hamiltonians*

$$\hat{\mathcal{H}}_{\text{doub}} = \begin{pmatrix} \frac{B_0}{2} g_{zz} & 0 \\ 0 & -\frac{B_0}{2} g_{zz} \end{pmatrix} \quad (1)$$

(columns labelled $|\alpha\rangle$ and $|\beta\rangle$)

*The unit of the magnetic field in the matrices is Hz.

$$
\hat{\mathcal{H}}_{\text{trip}} = \begin{pmatrix} B_0 g_{zz} + \frac{D_{zz}}{2} & 0 & 0 \\ 0 & -D_{zz} & 0 \\ 0 & 0 & -B_0 g_{zz} + \frac{D_{zz}}{2} \end{pmatrix} \quad (2)
$$

with columns labelled $|\mathrm{T}_+\rangle$, $|\mathrm{T}_0\rangle$, $|\mathrm{T}_-\rangle$.

$$
\hat{\mathcal{H}}_{\text{rp}} = \begin{pmatrix} \frac{B_0}{2}(g1_{zz} + g2_{zz}) + \frac{J}{2} - \frac{D_{zz}}{2} & 0 & 0 & 0 \\ 0 & -1/2J & \frac{B_0}{2}(g1_{zz} - g2_{zz}) & 0 \\ 0 & \frac{B_0}{2}(g1_{zz} - g2_{zz}) & 1/2J + D_{zz} & 0 \\ 0 & 0 & 0 & -\frac{B_0}{2}(g1_{zz} + g2_{zz}) + \frac{J}{2} - \frac{D_{zz}}{2} \end{pmatrix} \quad (3)
$$

with columns labelled $|\mathrm{T}_+\rangle$, $|\mathrm{S}\rangle$, $|\mathrm{T}_0\rangle$, $|\mathrm{T}_-\rangle$.

$$
\hat{\mathcal{H}}_{\text{tdp}} = \hat{\mathcal{H}}_{\text{doub}} \otimes \mathbf{E}_3 + \mathbf{E}_2 \otimes \hat{\mathcal{H}}_{\text{trip}} +
$$

$$
\begin{pmatrix} \frac{D_{zz}+J}{2} & 0 & 0 & 0 & 0 & 0 \\ 0 & 0 & 0 & \frac{1}{\sqrt{3}}\left(-\frac{D_{zz}}{2} + J\right) & 0 & 0 \\ 0 & 0 & -\frac{D_{zz}+J}{2} & 0 & \frac{1}{\sqrt{3}}\left(-\frac{D_{zz}}{2} + J\right) & 0 \\ 0 & \frac{1}{\sqrt{3}}\left(-\frac{D_{zz}}{2} + J\right) & 0 & -\frac{D_{zz}+J}{2} & 0 & 0 \\ 0 & 0 & \frac{1}{\sqrt{3}}\left(-\frac{D_{zz}}{2} + J\right) & 0 & 0 & 0 \\ 0 & 0 & 0 & 0 & 0 & \frac{D_{zz}+J}{2} \end{pmatrix} \quad (4)
$$

with columns labelled $|\alpha, \mathrm{T}_+\rangle$, $|\alpha, \mathrm{T}_0\rangle$, $|\alpha, \mathrm{T}_-\rangle$, $|\beta, \mathrm{T}_+\rangle$, $|\beta, \mathrm{T}_0\rangle$, $|\beta, \mathrm{T}_-\rangle$.

$$
\hat{\hat{\mathcal{H}}}_{\text{commutator superoperator}} = \mathbf{E} \otimes \hat{\mathcal{H}} - \hat{\mathcal{H}}^{\dagger} \otimes \mathbf{E} \quad (5)
$$

2.2 Relaxation

Phenomenological relaxation superoperator The relaxation superoperator $\hat{\hat{\mathbf{R}}}$ is a square matrix with the squared dimension of the system. The phenomenological superoperator with the longitudinal relaxation rate $r_1 = 1/T_1$ and the transverse relaxation rate $r_2 = 1/T_2$ is set up as follows:

$$
\begin{aligned} \hat{\hat{\mathbf{R}}}_{ii,jj} &= r_1 \\ \hat{\hat{\mathbf{R}}}_{ij,ij} &= r_2 \\ \hat{\hat{\mathbf{R}}}_{ii,ii} &= -\sum \hat{\hat{\mathbf{R}}}_{ii,jj}. \end{aligned} \quad (6)
$$

As an example, the matrix is given for a spin-1 system:

$$\hat{\hat{\mathbf{R}}} = \begin{pmatrix} -2r_1 & 0 & 0 & 0 & r_1 & 0 & 0 & 0 & r_1 \\ 0 & r_2 & 0 & 0 & 0 & 0 & 0 & 0 & 0 \\ 0 & 0 & r_2 & 0 & 0 & 0 & 0 & 0 & 0 \\ 0 & 0 & 0 & r_2 & 0 & 0 & 0 & 0 & 0 \\ r_1 & 0 & 0 & 0 & -2r_1 & 0 & 0 & 0 & r_1 \\ 0 & 0 & 0 & 0 & 0 & r_2 & 0 & 0 & 0 \\ 0 & 0 & 0 & 0 & 0 & 0 & r_2 & 0 & 0 \\ 0 & 0 & 0 & 0 & 0 & 0 & 0 & r_2 & 0 \\ r_1 & 0 & 0 & 0 & r_1 & 0 & 0 & 0 & -2r_1 \end{pmatrix}. \tag{7}$$

Limitations and advice on the supported magnitude of relaxation times Plausible results can be only obtained when the relaxation times are chosen in an appropriate way. The following can be taken as a guide:

- Relaxation times should be $> 10^{-8}$ s, as the spectra are very broad for very short times after the laser pulse.
- Relaxation rates should be $< 10^{8}$ s^{-1} for the same reason.
- One should choose $t \approx 2T_{\text{relax}}$ to see relaxation effects.
- If you see sharp bends in the spectrum at later times, this is due to a numerical error. In this case, you should increase the number of B-points.
- The best results are obtained for time constants around 10^{-6} s: The spectrum is neither too broad nor the required number of B-points too large.
- Using a user-defined relaxation matrix, only population changes within the spin system can be simulated. It is not possible to alter these states, meaning that any processes affecting the energy levels of the system, such as chemical reactions or conformational changes, cannot be simulated.

3 Experimental details for Figure 4

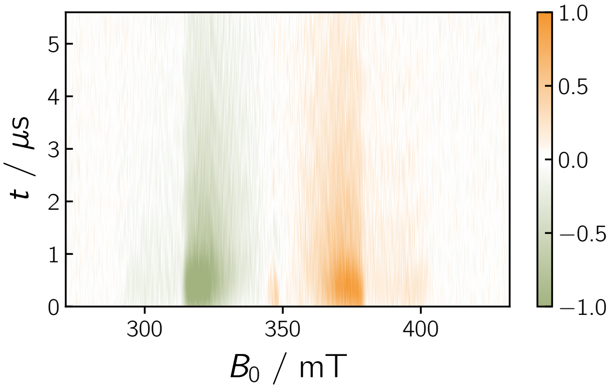

Figure S2: Experimental dataset corresponding to the simulation in Fig.4 in the main part.

Figure S2 shows the experimental data underlying the simulation in Fig.4 in the main part. The sample was prepared with an absorbance of roughly 0.3 at the excitation wavelength of 435 nm, measured in a 2 mm cuvette. The sample solution was then transferred into quartz EPR tubes with an outer diameter of 3.8 mm (inner diameter of 3 mm). The solutions were rapidly frozen in liquid nitrogen before insertion into the EPR resonator for the measurement. The transient continuous wave EPR measurement was performed at the X-band (9.75 GHz) on a

Bruker ELEXSYS E580 spectrometer at a constant temperature of 80 K, using an Oxford Instruments nitrogen gas-flow cryostat (CF 935). The sample was excited directly with depolarized laser light through the optical window of the cryostat and resonator. An excitation energy of ~2.3 mJ was used.

The data were acquired in direct detection mode with a video amplifier bandwidth of 20 MHz using the built-in transient recorder and a microwave power of 1.5 mW (20 dB). The transient recorder was used in AC-AFC mode and the transient signal was fed through a low-noise voltage preamplifier (Stanford Research Systems SR 560) with a bandpass filter of 3 kHz−1 MHz before entering the detection circuitry. For every magnetic field value, a time trace with 4096 points was recorded using a time base of 4 ns. After data acquisition, the 2D spectrum was baseline-corrected in both dimensions using a lab-written MATLAB routine, frequency-corrected to 9.75 GHz and field-corrected using a carbon fibre standard with $g = 2.002644$.[1]

4 Simulation parameters

Table S2: Parameters used in the simulations. See the Listings for more detailed information.

Figure 4	
spin system	"tdp"
g_r	$[2.0103, 2.0070, 2.0025]$
g_t	2.00522
D_{ZFS}	-1681 MHz
E_{ZFS}	40 MHz
J	400000 MHz
D	240 MHz
E	-50 MHz
precursor	"triplet-zf"
population	$[0.200, 0.201, 0.453, 0.358, 0.437]$
dynamics	$\begin{pmatrix} 0 & 7 & 0 & 0 & 0 & 0 \\ 7 & 0 & 0 & 0 & 0 & 0 \\ 0 & 0 & 0 & 1.5 & 0 & 7 \\ 0 & 0 & 1.5 & 0 & 7 & 0 \\ 0 & 0 & 0 & 7 & 0 & 1.5 \\ 0 & 0 & 7 & 0 & 1.5 & 0 \end{pmatrix} \frac{1}{\mu s}$
Figure 5	
spin system	"doublet"
g	2
precursor	"eigen"
population	$[1, 0]$
decay	5 μs
Figure 6	
spin system	"rp"
g_1	$[2.00304, 2.00262, 2.00232]$
g_1 Frame	$[-0.262, 0.489, 0.471]$
g_2	$[2.00564, 2.00494, 2.00217]$
precursor	"singlet"
D	1.121 MHz
D Frame	$[0, 1.012, -0.017]$
decay	1 μs
B_{mw}	0.03 mT
Figure 7 and 8	
spin system	"trip"
g	2
D	901
E	-164
precursor	"zf"
population	$[0.86, 0, 0.14]$

Figure 9

spin system	"tdp"
g_r	2.0059
g_t	2.003
D_{ZFS}	700 MHz
J	−20000 MHz
precursor	"eigen"
population	[0.3, 0.225, 0.2, 0.3, 0.5, 0.48]
dynamics	$\begin{pmatrix} 0 & 0 & 0 & 0 & 206 & 133 \\ 0 & 0 & 0 & 0 & 0 & 69 \\ 0 & 0 & 0 & 0 & 267 & 0 \\ 0 & 0 & 0 & 0 & 3038 & 802 \\ 206 & 0 & 267 & 802 & -250 & 0 \\ 133 & 69 & 0 & 802 & 0 & -250 \end{pmatrix} \frac{10^{-3}}{\mathrm{s}}$

Figure 10

spin system	"rp"
g_1	[2.00304, 2.00262, 2.00232]
g_1 Frame	[−0.262, 0.489, 0.471]
g_2	[2.00564, 2.00494, 2.00217]
precursor	"singlet"
D	−3.363 MHz
D Frame	[0, 1.012, −0.017]
decay	7.5 μs
spin system	"trip"
g	2.008
D	898
E	-161
precursor	"zf"
population	[0.95, 0, 0.05]
dynamics	$\begin{pmatrix} 0 & 0.25 & 0 \\ 0.25 & 0 & 0.01 \\ 0 & 0.01 & 0 \end{pmatrix} \frac{1}{\mu\mathrm{s}}$

5 Source code of the simulations

Listing S1: Source code for the simulation of the spectrum in Fig.4. The four steps to the full spectrum are indicated.

```
import teacups.simulations as sim
import teacups.classes as cl
import numpy as np

# %% Step 1: Determination of the spin system

Sys = cl.Sys()
Exp = cl.Exp()
SimOpt = cl.SimOpt()


# choose a triplet doublet pair spin system
```

```
Sys.spin_system = "tdp"

# set up g-tensors of the radical and the triplet
Sys.g = [2.0103, 2.0070, 2.0025]
Sys.g_tri = [2.00522, 2.00522, 2.00522]

# define triplet ZFS
Sys.D_tri = -1681
Sys.E_tri = 40

# define couplings of the radical and the triplet
Sys.J_ex = 400000
Sys.D = 160*1.5
Sys.E = -50

# define initial state
Sys.precursor = "triplet-zf"
Sys.population = [0.2, 0.201, 0.4529, 0.3578, 0.4373]

# define decay time and line width
Sys.decay = 3e-6
Sys.width_gauss = 6

# experimental setup
Exp.B_z = np.linspace(271.492, 432.075, 1000)
Exp.t_scale = [0, 1e-6]
Exp.t_points = 2
Exp.B_mw = 0.001
Exp.freq_mw = 9.75e9

# simulation options
SimOpt.grid = "sophe"
SimOpt.sym = "D2h"
SimOpt.grid_points = 20
SimOpt.space = "hilbert"
SimOpt.eigval_mode = False

# do the simulation
spc = sim.teacups(Sys, Exp, SimOpt)

# %% Step 2: Analysis of the energy of the eigenstates

SimOpt.eigval_mode = True
eigenvalues = sim.teacups(Sys, Exp, SimOpt)


# %% Step 3: Set up the dynamics

k_ic = 7/1e-6
k_isc = 1.5/1e-6

R = np.zeros((6, 6))

# Doublet
R[0, 1] = k_ic
R[1, 0] = k_ic

# +- 3/2
R[2, 5] = k_ic
R[5, 2] = k_ic

# +- 1/2
R[3, 4] = k_ic
R[4, 3] = k_ic

# +-3/2 <-> +- 1/2
R[2, 3] = k_isc
R[3, 2] = k_isc

R[4, 5] = k_isc
R[5, 4] = k_isc
```

```
Sys.dynamics = R

# Alternative for phenomenological relaxation superoperator
# Sys.T_relax_1 = 1e-6
# Sys.T_relax_2 = 1e-6


# %% Liouville-space simulation

SimOpt.eigval_mode = False
SimOpt.space = "liouville"
SimOpt.pop_evolution = True
Exp.t_points = 100

# Run the simulation
spc, pop_evolution = sim.teacups(Sys, Exp, SimOpt)
```

Listing S2: Source code for the simulation of the transient nutations in doublet spectra in Fig.5.

```
import teacups.simulations as sim
import teacups.classes as cl
import numpy as np

# initialize classes with default parameters
Sys = cl.Sys()
Exp = cl.Exp()
SimOpt = cl.SimOpt()

# set up spin System parameters
Sys.g = [2, 2, 2]
Sys.width_gauss = 3
Sys.decay = 5e-6

Sys.spin_system = 'doub'
Sys.precursor = 'eigen'
Sys.population = [1, 0]

# set up Experimental parameters
Exp.B_z = np.linspace(320, 380, 3000)
Exp.t_scale = [0, 10e-6]
Exp.t_points = 100
Exp.B_mw = 0.2 # change for other two simulations to 0.05 and 0.1

# set up simulation SimOption parameters
SimOpt.grid_points = 15
SimOpt.space = 'hilbert'
SimOpt.cpu_cores = 0

# do simulation
spec = sim.teacups(Sys, Exp, SimOpt)
spec = spec/abs(spec).real.max()
```

Listing S3: Source code for the simulation of the zero quantum coherence in a radical pair spectrum in Fig.6.

```
import teacups.simulations as sim
import teacups.classes as cl
import numpy as np

# initialize classes with default parameters
Sys = cl.Sys()
Exp = cl.Exp()
SimOpt = cl.SimOpt()

Sys.spin_system = 'rp'
Sys.g1 = [2.00304, 2.00262, 2.00232]
Sys.g1_frame = [-0.262, 0.489, 0.471]
Sys.g2 = [2.00564, 2.00494, 2.00217]

Sys.precursor = 'singlet'

```

```
Sys.decay = 1e-6

Sys.D = 1.131
Sys.D_frame = [0, 1.012, -0.017]
Sys.J_ex = 0

Sys.width_gauss = 0.08

Exp.B_z = np.linspace(350.5, 352.3, 800)
Exp.t_scale = [0, 2e-6]
Exp.t_points = 300
Exp.B_mw = 0.03
Exp.freq_mw = 9.8562*1e9

SimOpt.grid_points = 20
SimOpt.space = 'hilbert'

# do simulation
spec = sim.teacups(Sys, Exp, SimOpt)
spec = spec/abs(spec).real.max()
```

Listing S4: Source code for the simulation of the relaxation time dependence of a triplet spectrum in Fig.7.

```
import teacups.simulations as sim
import teacups.classes as cl
import numpy as np

# initialize classes with default parameters
Sys = cl.Sys()
Exp = cl.Exp()
SimOpt = cl.SimOpt()

# set up spin System parameters
Sys.g_tri = [2, 2, 2]
Sys.D_tri = 901
Sys.E_tri = -164

Sys.spin_system = 'trip'
Sys.precursor = 'zf'
Sys.population = [0.86, 0, 0.14]

Sys.T_relax_1 = 5e-6 # changed to 1e-6
Sys.T_relax_2 = 5e-6 # changed to 0.1e-6

Sys.width_gauss = 3

# set up Experimental parameters
Exp.B_z = np.linspace(300, 400, 1000)
Exp.t_scale = [0, 2e-6]
Exp.t_points = 100

# set up simulation SimOption parameters
SimOpt.grid_points = 21
SimOpt.space = 'liouville'
SimOpt.pop_evolution = True


# do simulation
spec, pop_evolution = sim.teacups(Sys, Exp, SimOpt)
spec = spec/abs(spec).real.max()
```

Listing S5: Source code for the simulation of the asymmetric relaxation of a triplet spectrum in Fig.8.

```
import teacups.simulations as sim
import teacups.classes as cl
import numpy as np

# initialize classes with default parameters
Sys = cl.Sys()
Exp = cl.Exp()
```

```
SimOpt = cl.SimOpt()

# set up spin System parameters
Sys.g_tri = [2, 2, 2]
Sys.D_tri = 901
Sys.E_tri = -164

Sys.spin_system = 'trip'
Sys.precursor = 'zf'
Sys.population = [0.86, 0, 0.14]

Sys.dynamics = np.array(
    [[0, 1/1e-6, 1/2e-6], [1/1e-6, 0, 1/0.5e-6], [1/2e-6, 1/0.5e-6, 0]])

Sys.width_gauss = 3

# set up Experimental parameters
Exp.B_z = np.linspace(300, 400, 1000)
Exp.t_scale = [0, 2e-6]
Exp.t_points = 100

# set up simulation SimOption parameters
SimOpt.grid_points = 21
SimOpt.space = 'liouville'
SimOpt.pop_evolution = True

# do simulation
spec, pop_evolution = sim.teacups(Sys, Exp, SimOpt)
spec = spec/abs(spec).real.max()
```

Listing S6: Source code for the simulation of the reverse quartet mechanism in a triplet–doublet pair spectrum in Fig.9.

```
import teacups.simulations as sim
import teacups.classes as cl
import numpy as np
import matplotlib.pyplot as plt
import time as time

name = "tdp_rqm"

Sys = cl.Sys()
Exp = cl.Exp()
SimOpt = cl.SimOpt()

# choose a triplet doublet pair spin system
Sys.spin_system = "tdp"

# set up g-tensors of the radical and the triplet
Sys.g = [2.0059, 2.0059, 2.0059]
Sys.g_tri = [2.003, 2.003, 2.003]

# define triplet ZFS
Sys.D_tri = 700

# define couplings of the radical and the triplet
Sys.J_ex = -20000

# define initial state
Sys.precursor = "eigen"
Sys.population = [0.3, 0.225, 0.2, 0.3, 0.5, 0.48]

# define line width
Sys.width_gauss = 1

# experimental setup
Exp.B_z = np.linspace(320, 380, 700)
Exp.t_scale = [0, 2e-6]
Exp.t_points = 60
Exp.B_mw = 0.001
Exp.freq_mw = 9.75e9

```

```
# simulation options
SimOpt.grid_points = 7
SimOpt.space = "liouville"
SimOpt.pop_evolution = True

# %% set up dynamics-matrix

# determine eigenvalues
SimOpt.eigval_mode = True
eigval = sim.teacups(Sys, Exp, SimOpt)
e = np.mean(eigval[350], axis=0)

# build delta E between trip-doublet and trip-quartet states
de_53 = e[5]-e[3]
de_51 = e[5]-e[1]
de_50 = e[5]-e[0]
de_43 = e[4]-e[3]
de_42 = e[4]-e[2]
de_40 = e[4]-e[0]

# Dipolar coupling squared
D = (Sys.D_tri*1e6)**2

# set isc and doublet decay rates
k_isc = 0.3/1e-11
k_d = 0.25/1e-6

# set up dynamics matrix
R = np.zeros((6, 6))
R[5, 5] = -k_d
R[4, 4] = -k_d

R[5, 3] = k_isc/45*(D/(de_53)**2)
R[5, 1] = k_isc/135*(D/(de_51)**2)
R[5, 0] = k_isc/45*(D/(de_50)**2)
R[3, 5] = k_isc/45*(D/(de_53)**2)
R[1, 5] = k_isc/135*(D/(de_51)**2)
R[0, 5] = k_isc/45*(D/(de_50)**2)

R[4, 3] = k_isc/45*(D/(de_42)**2)
R[4, 2] = k_isc/135*(D/(de_42)**2)
R[4, 0] = k_isc/45*(D/(de_40)**2)
R[3, 4] = k_isc/45*(D/(de_43)**2)
R[2, 4] = k_isc/135*(D/(de_42)**2)
R[0, 4] = k_isc/45*(D/(de_40)**2)

Sys.dynamics = R

# %%
SimOpt.eigval_mode = False

# do simulation
spec, pop_evolution = sim.teacups(Sys, Exp, SimOpt)
spec = spec/abs(spec).real.max()
```

Listing S7: Source code for the simulation of the time resolved spectrum of PS I in Fig.10.

```
import numpy as np
import teacups.simulations as sim
import teacups.classes as cl


# initialize classes with default parameters
Sys = cl.Sys()
Exp = cl.Exp()
SimOpt = cl.SimOpt()

# set up spin system parameters
Sys.spin_system = 'rp'
Sys.g1 = [2.00304, 2.00262, 2.00232]
Sys.g2 = [2.00564, 2.00494, 2.00217]
```

```
Sys.g1_frame = np.array([-15, 28, 27])*(np.pi/180)

Sys.precursor = 'singlet'

Sys.decay = 0.75e-7
Sys.T_relax_1 = 2e-6
Sys.T_relax_2 = 500e-9

Sys.D = -3.3630
Sys.D_frame = np.array([0, 58, -1])*(np.pi/180)
Sys.J_ex = 0

Sys.width_gauss = 0.125

# set up experimental parameters
Exp.B_z = np.linspace(350.55, 352.33, 500)
Exp.t_scale = [0, 100e-9]
Exp.t_points = 11
Exp.B_mw = 0.03
Exp.freq_mw = 9.8562*1e9

# set up simulation option parameters
SimOpt.grid_points = 20
SimOpt.space = 'hilbert'

# do the simulation
spec = sim.teacups(Sys, Exp, SimOpt)
t = np.linspace(Exp.t_scale[0], Exp.t_scale[1], Exp.t_points)
B = Exp.B_z
```

Listing S8: Source code for the simulation of the time resolved spectrum of a Zn-porphyrine monomer in Fig.10.

```
import teacups.simulations as sim
import teacups.classes as cl
import numpy as np


# initialize classes with default parameters
Sys = cl.Sys()
Exp = cl.Exp()
SimOpt = cl.SimOpt()

# set up spin system parameters
Sys.g_tri = [2.008, 2.008, 2.008]
Sys.D_tri = 898
Sys.E_tri = -161

Sys.spin_system = 'trip'
Sys.precursor = 'zf'
Sys.population = [0.95, 0, 0.05]

Sys.decay = 1e-6
Sys.dynamics = np.array([[0, 0.25e6, 0],
                         [0.25e6, 0, 0.01e6],
                         [0, 0.01e6, 0]])
Sys.width_gauss = 2

# set up experimental parameters
Exp.B_z = np.linspace(295.15595, 394.20545, 1024)
Exp.t_scale = [1.9e-6, 4.996e-6]
Exp.t_points = 775
Exp.B_mw = 0.0001

# set up simulation option parameters
SimOpt.grid_points = 40
SimOpt.grid = 'sophe'
SimOpt.sym = "D2h"
SimOpt.space = 'liouville'
SimOpt.cpu_cores = 0

# do simulation
```

```
spec = sim.teacups(Sys, Exp, SimOpt)

# cut spectrum to the experimental starting point at t=2e-6 s
spec = spec[25:].real/max(abs(spec[25].real))
```

6 Programming details

The entire code of `teacups`, including a detailed documentation, can be found at:
`https://github.com/TheresiaQuintes/teacups`.

Some details on the programming are given below to help users find a starting point. Here, we only give a short overview of the code and an introduction to the main documentation. For further details, the reader is referred to the main documentation (link above).

`teacups` consists of 13 Python modules, which contain classes and functions. They can be sorted roughly into three categories, namely specific simulation modules, general modules, and structuring modules.

6.1 Specific simulation modules

These modules include all functions that are specific for the simulation of transient EPR spectra:

- creators.py
- density_matrices.py
- hamiltonians.py
- hyperfine.py
- input_handler.py
- relaxation.py
- signals_and_processing.py
- simulations.py.

The main file simulations.py calls all functions in the right order to obtain the desired spectrum. The other modules are used to set up and transform the explicit matrices as described in the theory part. The functions of the specific simulation modules use a four-class system as input and output, which consists of `Sys`, `Exp`, `SimOpt` and `Cal`. All information needed for the simulation is provided and sorted in these four classes as attributes:

- `Sys` contains all spin system parameters including relaxation times.
- `Exp` contains all experimental parameters.
- `Opt` contains simulations options, e.g. the choice of the space, the number of grid points, and the simulation mode.

6.2 General modules

These modules contain functions that are generally usable (and could be used outside of `teacups`):

- convolution.py
- grid.py
- orientation_dependent_ham.py

- relaxation.py.

Included are, amongst others, the setup of a grid on a sphere, the Gaussian convolution of a spectrum, or the general setup of a relaxation matrix/ Hamiltonian. The functions take parameters as input and return the desired output. They do not use the four-class system, which makes them more flexible.

6.3 Structuring modules

All matrices of the specific simulation modules are objects of the classes defined in the structuring modules:

- matrix_tools.py
- multioperator_tools.py.

Both classes define matrices including methods like rotation to a number of angle combinations, the setup of a Hamiltonian etc. An object of class `multioperator` has an attribute called `B_angle_matrix`. This is a three-dimensional `NumPy` array containing the values of the operator for a number of magnetic field points and a number of angle points. Its dimension is `len(B)` × `len(angles)` × `spinsystem dimension` × `spinsystem dimension`. The specific simulation modules often use objects of multioperator-type, which has the advantage, that calculations can be performed for all field points and angles at once without using loops. This makes the code much faster.

7 Code optimization

The calculation of the time-evolution of an experimental spectrum is computationally rather expensive. Thus, some effort has been invested to optimize the performance of the code. In the following, an overview of the optimization attempts is given:

- **Vectorization using NumPy[2]:** Using the multioperator-class (as described above) all calculations are performed simultaneously since the matrices are collected in one big array. This has great advantages as nearly no loops are needed and fast built-in NumPy functions (partly based on efficient Fortran routines from LAPACK[3]) are used. This comes with the disadvantage that more memory is needed to save the big NumPy-arrays.
- **Parallel computing:** The NumPy functions automatically use multiple cores, which works well. Further, parallel computing is applied to the time-evolution loop. The array is split along the magnetic field axis and the evolution for multiple magnetic field points is done simultaneously.
- **Numba[4]:** Using just-in-time-compilation with Numba could increase the performance of Python-loops. As most of the code is vectorized, no loops are needed and Numba could only be used for the time-evolution loop. It should be noted that Numba does not work in combination with SciPy (see below).
- **The matrix exponential:** The bottleneck of the simulations is the calculation of the matrix exponential. Using NumPy, it can be calculated by diagonalising the exponents matrix. For this purpose, the function numpy.eig is used. The faster function numpy.eigh cannot be used as the matrix is not Hermitian. NumPy uses Fortran LAPACK routines for the diagonalization and already works rather efficiently. Nevertheless, we tried to speed up the calculations.
- **SciPy[5]:** For the calculation of the matrix exponential, the SciPy function works more efficiently than the NumPy function. However, using SciPy, vectorization cannot be used and loops are needed for magnetic field points and angle points. The latter cannot be accelerated using Numba as Numba does not work in combination with SciPy. Benchmarks showed that the use of NumPy without loops is faster than the use of SciPy with loops.

- **Julia[6]:** As the loops seemed to be the problem when using SciPy, Julia-code has been integrated for the calculation of the matrix exponential. Due to just-in-time-compilation, loop-overlays are faster in Julia than in Python. Nevertheless, the performance could not be optimized since the matrix exponential function in Julia also uses the Fortran LAPACK routine. Consequently, the calculation was found to be just as fast using Julia as compared to NumPy.

- **FastExpm[7,8] and Expokit[9]:** In Julia, some packages for the fast calculation of matrix exponentials are available. However, the maximum matrix size of 36x36 for a triplet-douplet pair in Liouville space is still to small to achieve a significant improvement in performance using these functions.

- **Using the GPU:** For Python the `CuPy`[10] package may be used to calculate expensive matrix algebra on the GPU. For some basis transformations for triplet doublet pairs this is already implemented. Until now, no function for non-hermitean matrix-exponentials exists. They are announced for the next version of `CuPy`. As soon as the function is available, calculations on the GPU could make the code significantly faster.

References

[1] Herb, K.; Tschaggelar, R.; Denninger, G.; Jeschke, G. Double resonance calibration of *g* factor standards: carbon fibers as a high precision standard. *J. Magn. Reson.* **2018**, *289*, 100–106.

[2] Harris, C. R. et al. Array programming with NumPy. *Nature* **2020**, *585*, 357–362.

[3] Anderson, E.; Bai, Z.; Bischof, C.; Blackford, S.; Demmel, J.; Dongarra, J.; Du Croz, J.; Greenbaum, A.; Hammarling, S.; McKenney, A.; Sorensen, D. *LAPACK users' guide*, 3rd ed.; Society for Industrial and Applied Mathematics: Philadelphia, PA, 1999.

[4] Lam, S. K.; Pitrou, A.; Seibert, S. Numba: a LLVM-based python JIT compiler. Proceedings of the Second Workshop on the LLVM Compiler Infrastructure in HPC. New York, NY, USA, 2015.

[5] Virtanen, P. et al. SciPy 1.0: Fundamental algorithms for scientific computing in python. *Nat. Methods* **2020**, *17*, 261–272.

[6] Bezanson, J.; Edelman, A.; Karpinski, S.; Shah, V. B. Julia: A fresh approach to numerical computing. *SIAM review* **2017**, *59*, 65–98.

[7] Hogben, H.; Krzystyniak, M.; Charnock, G.; Hore, P.; Kuprov, I. Spinach – A software library for simulation of spin dynamics in large spin systems. *J. Magn. Reson.* **3022**, *208*, 179–194.

[8] Kuprov, I. Diagonalization-free implementation of spin relaxation theory for large spin systems. *J. Magn. Reson.* **2011**, *209*, 31–38.

[9] Sidje, R. B. Expokit: A software package for computing matrix exponentials. *ACM Trans. Math. Softw.* **1998**, *24*, 130–156.

[10] Okuta, R.; Unno, Y.; Nishino, D.; Hido, S.; Loomis, C. CuPy: A NumPy-Compatible Library for NVIDIA GPU Calculations. Proceedings of Workshop on Machine Learning Systems (LearningSys) in The Thirty-first Annual Conference on Neural Information Processing Systems (NIPS). 2017.
